# Three dominant awnless genes in common wheat: Fine mapping, interaction and contribution to diversity in awn shape and length

**DOI:** 10.1371/journal.pone.0176148

**Published:** 2017-04-24

**Authors:** Motohiro Yoshioka, Julio C. M. Iehisa, Ryoko Ohno, Tatsuro Kimura, Hiroyuki Enoki, Satoru Nishimura, Shuhei Nasuda, Shigeo Takumi

**Affiliations:** 1 Graduate School of Agricultural Science, Kobe University, Kobe, Japan; 2 Departamento de Biotecnología, Facultad de Ciencias Químicas, Universidad Nacional de Asunción, San Lorenzo, Paraguay; 3 Graduate School of Science, Technology and Innovation, Kobe University, Kobe, Japan; 4 Biotechnology & Afforestation Laboratory, New Business Planning Division, TOYOTA Motor Corporation, Miyoshi, Aichi, Japan; 5 Frontier Research Planning Department, Frontier Research Center, TOYOTA Motor Corporation, Toyota, Aichi, Japan; 6 Graduate School of Agriculture, Kyoto University, Kyoto, Japan; Western Australia Department of Agriculture and Food, AUSTRALIA

## Abstract

The awn is a long needle-like structure formed at the tip of the lemma in the florets of some grass species. It plays a role in seed dispersal and protection against animals, and can contribute to the photosynthetic activity of spikes. Three main dominant inhibitors of awn development (*Hd*, *B1* and *B2*) are known in hexaploid wheat, but the causal genes have not been cloned yet and a genetic association with awn length diversity has been found only for the *B1* allele. To analyze the prevalence of these three awning inhibitors, we attempted to predict the genotypes of 189 hexaploid wheat varieties collected worldwide using markers tightly linked to these loci. Using recombinant inbred lines derived from two common wheat cultivars, Chinese Spring and Mironovskaya 808, both with short awns, and a high-density linkage map, we performed quantitative trait locus analysis to identify tightly linked markers. Because this linkage map was constructed with abundant array-based markers, we converted the linked markers to PCR-based markers and determined the genotypes of 189 hexaploids. A significant genotype-phenotype correlation was observed at the *Hd* and *B1* regions. We also found that interaction among these three awning inhibitors is involved in development of a membranous outgrowth at the base of awn resembling the *Hooded* mutants of barley. For the hooded awn phenotype, presence of the *Hd* dominant allele was essential but not sufficient, so *B2* and other factors appear to act epistatically to produce the ectopic tissue. On the other hand, the dominant *B1* allele acted as a suppressor of the hooded phenotype. These three awning inhibitors largely contribute to the genetic variation in awn length and shape of common wheat.

## Introduction

The awn is a long needle-like structure formed on the distal end of the lemma in the florets of some grass species such as wheat, barley and rice. This extension of the lemma seems to be a modified leaf blade [1] and can serve to protect against animals [2]. The presence of silicified hairs on its surface facilitates seed dispersal by adhesion to animal fur [3]. The dispersal unit of wild wheat bears two pronounced awns that balance each unit as it falls, and the movements of the two awns, driven by the daily humidity cycle, propel the seeds into the soil [4].

Awns also contribute to the photosynthetic activity of the inflorescence in wheat and barley. In tetraploid wheat (*Triticum turgidum* L. subsp. *durum*) the total surface area of the awns can exceed that of the leaf blade [5], and more than half of the total number of stomata of wheat spikelets is present in the awns [6]. Because the pathway for assimilate movement from awns to the kernels is minimal, the awns can be considered as an ideal place for light interception and CO_2_ uptake [7]. These observations may contribute to the observation that the presence of awns can double the net rate of spike photosynthesis [8]. Although awns have only a limited effect on yield in wet climates, their contribution is significantly higher than in awnless or de-awned wheat varieties in drier conditions [9].

During domestication of rice, humans have selected awnless varieties because of the convenience of seed collection and the ease of seed storage. In contrast to barley and wheat, rice awns do not seem to contribute to the photosynthetic activity of the panicle, since they have no chlorenchyma [10]. Many genes involved in awn formation and elongation have been identified in rice, such as a basic helix-loop-helix transcription factor named *Awn-1* (*An-1*) [11], a YABBY transcription factor (*DROOPING LEAF* [*DL*]) [12], an auxin responsive factor, *OsETTIN2* [12], *LONG AND BARBED AWN1* (*LABA1*), encoding a cytokinin-activating enzyme [13], and *REGULATOR OF AWN ELONGATION 2* (*RAE2*), which is a member of the EPIDERMAL PATTERNING FACTOR-LIKE (EPFL) family [14].

In barley, *short awn 2* (*Lks2*), located on chromosome 7H and encoding a *SHORT INTERNODES* (*SHI*) family transcription factor, has been identified as a causal gene for short awns in Eastern Asian accessions [15]. The dominant *Hooded* mutation (*K*) is also known in barley, transforming the awns into an extra flower of inverse polarity on the lemma, which is caused by a 305 bp duplication in the homeobox gene *HvKnox3* [16,17].

In hexaploid common wheat (*Triticum aestivum* L.), three dominant inhibitors of awn development are known as *Hooded* (*Hd*), *Tipped1* (*B1*) and *Tipped2* (*B2*) [18,19], which are respectively located on chromosome arms 4AS, 5AL and 6BL [20]. In wheat varieties with the dominant *Hd* allele, awns are reduced in length and are curved and twisted near the base. In some cases, the awn is considerably broadened at the base and could possess membranous lateral expansions, resembling the *Hooded* mutants of barley. The dominant *B1* mutation produces short awns at the base and middle of the spike, but the length increases toward the top of the spike and may reach 1 cm. These awn tips are usually straight and unbent at the base. In contrast, in *B2* mutants, the awn length is nearly equal all along the spike, and the longest ones are found near the middle of spike. The *B2* mutant awns are often gently curved but are never bent around themselves as in *Hd*, and do not produce membranous lateral outgrowths [18]. Wheat varieties homozygous for the three recessive *hd*, *b1* and *b2* alleles are fully awned, while those with two dominant inhibitors such as *HdHdb1b1B2B2* and *hdhdB1B1B2B2* are awnless [19]. Using a doubled haploid population derived from two common wheat varieties, Chinese Spring (CS) with the *HdHdb1b1B2B2* awnless genotype and Courtot with the *hdhdb1b1b2b2* fully awned genotype, quantitative trait locus (QTL) analysis was performed for awn length, and the chromosomal locations of *Hd* and *B2* were determined [21]. *B1* is a phenotypic marker on wheat genetic maps [22–24]. In a previous association study using a panel of hexaploid wheat mainly composed of UK varieties, the presence of the dominant *B1* allele was found [25]. However, the prevalence of *Hd* and *B2* alleles in hexaploid wheat varieties was unknown.

In our previous study, an array-based genotyping system was developed and a high-density genetic map was constructed using recombinant inbred lines (RILs) derived from a cross between common wheat varieties CS and Mironovskaya 808 (M808) [26]. M808 presents a phenotype similar to that of the *B1* mutant, with extensive short awns at the base and middle of the spike but longer awns at the top. Using this mapping population, we aimed to identify markers tightly linked to the three awning inhibitor loci by performing QTL analysis and to then convert the linked array-based markers to PCR-based markers for genotyping 189 hexaploid wheat varieties to predict the presence of the *Hd*, *B1* and *B2* alleles. We also performed QTL of the membranous lateral outgrowth formation (hooded phenotype). The observed genetic interactions among the awning inhibitors and their contribution to awn shortening and the hooded phenotype are discussed.

## Materials and methods

### Plant materials and awn length evaluation

A mapping population of 210 RILs derived from a cross between two common wheat cultivars, CS and M808, developed by Kobayashi et al. [27], and 189 of the 192 hexaploid wheat lines (S1 Table) of the National BioResource Project (NBRP)’s hexaploid wheat core collection [28] (http://shigen.nig.ac.jp/wheat/komugi/) were used in this study. The plants were grown individually in pots arranged randomly in a field of Kobe University (348430N, 1358130E) in the 2012–13 season for RILs and the 2013–14 season for the hexaploid wheat core collection. Total DNA from each RIL and its parents was extracted from leaves using standard procedures.

**Fig 1:**
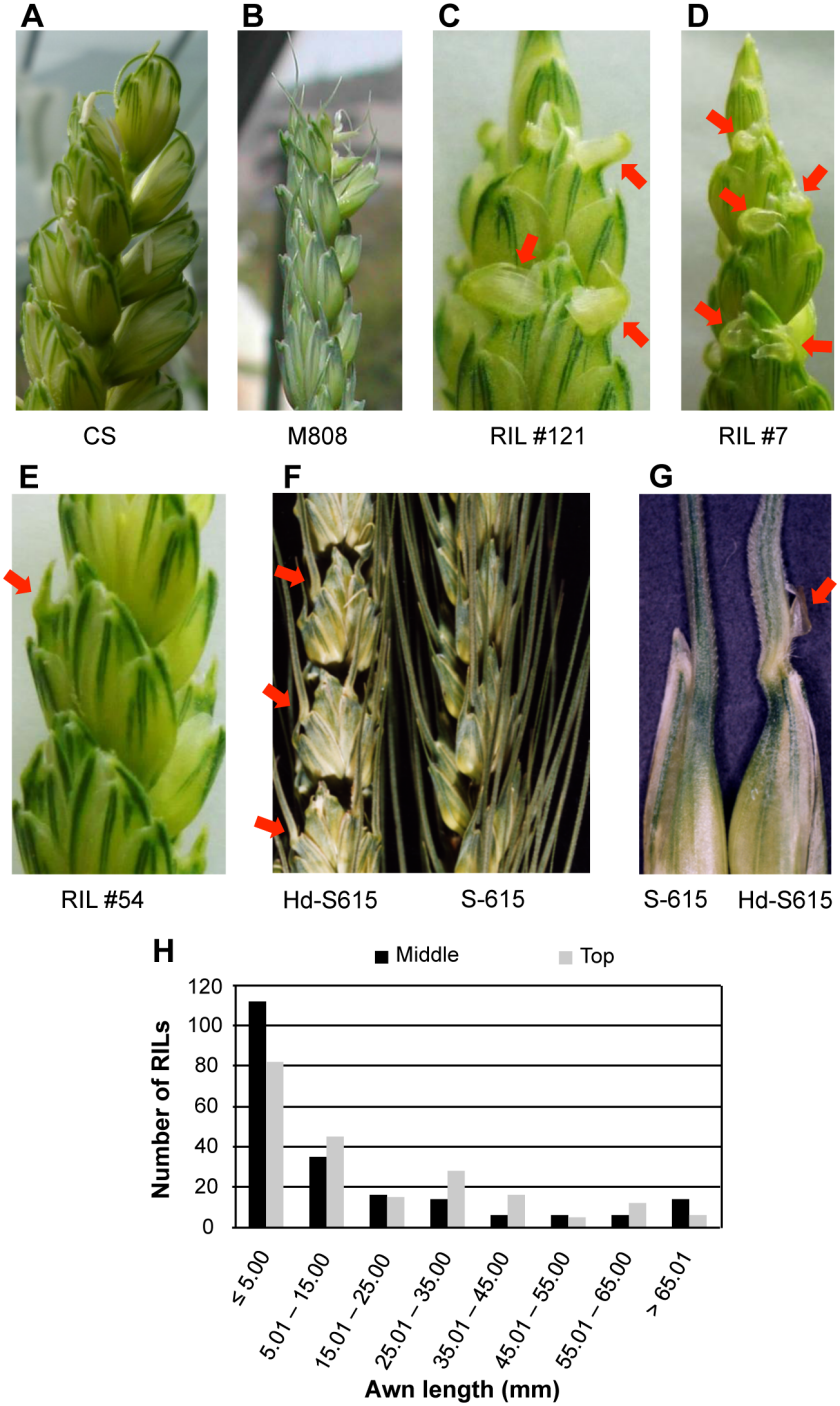
Awn phenotypes observed in the RIL population and near-isogenic line (NIL) of S-615. (A, B) Awns of the parental CS (A) and M808 (B) lines. (C-E) A hooded phenotype, defined as the presence of a membranous structure (C and D) or broadening of the base of awn (E) (indicated by red arrows), could be observed in some RILs but not in the parents. (F, G) Awn phenotype of a NIL of common wheat cv. S-615 with the *Hd* allele of CS (*Hd*-S615). The genotype of S-615 is *hdhdb1b1b2b2*, with long awns. (H) Frequency distribution of awn length at the middle (black bars) and top (grey bars) of the spike.

The awn length of the three main spikes of one plant for RILs and two plants for the hexaploid collection was measured at the top and middle of the spike at flowering. We also evaluated the hooded phenotype (Fig 1C and 1D) as the presence (trait value of 1 for QTL analysis) or absence (trait value of 0) of a membranous structure at the base of the awn. Some RILs also presented a broadening of the base of the awn (Fig 1E), and they were also considered as hooded for QTL analysis.

### QTL analysis

QTLs were analyzed by composite interval mapping using R/qtl package version 1.21–230, and the high-density linkage map had been previously constructed using the RIL population derived from CS and M808 [26]. First, the QTL genotype probability was calculated using the function calc.genoprob with a step size of 1 cM and the Kosambi map function. QTL analysis was performed with the cim function using three marker covariates and the Kosambi map function. The log-likelihood (LOD) score threshold was determined by computing a 1,000 permutation test for all traits. A multiple QTL model and epistasis were evaluated using the fitqtl function and the QTL locations were refined using the refineqtl function. The percentage of phenotypic variation (PV) explained by a QTL for a trait and epistasis were estimated using the fitqtl function in R/qtl.

### Conversion of array-based markers to PCR-based markers

For conversion to PCR-based markers, the read or contig sequences of the array-based markers located around the peak of the QTLs were used as a query to perform a BlastN search against the survey sequence of common wheat (S1 Fig) [26, 29]. The read or contig sequences of the array-based markers with the corresponding blast hit were aligned to search for putative *Pst*I and/or *Bst*NI sites. If no *Pst*I or *Bst*NI sites were observed, single nucleotide polymorphisms (SNPs) and indels (insetions/deletions) between CS and M808 were used to develop cleaved amplified polymorphic sequence (CAPS) markers. If neither *Pst*I/*Bst*NI sites nor SNPs were found, simple sequence repeat (SSR) motifs were searched for using SciRoKo ver. 3.4 [30]. Primers were designed using the Primer3Plus module (S2 Table) [31]. Electrophoresis of the PCR products and digested fragments was performed in 2% agarose gels for CAPS and indel markers, and 13% non-denaturing polyacrylamide gels were used for SSR markers. For polyacrylamide gel electrophoresis, the high efficiency genome scanning system (Nippon Eido, Tokyo, Japan) of Hori et al. [32] was used. Genotyping of RILs and the wheat core collection were performed using the markers developed. A linkage map was constructed using 161 RILs with predicted genotypes at the *Hd*, *B1* and *B2* loci and MapDisto ver. 1.7.7 software [33], and drawn by MapChart ver. 2.30 software [34].

## Results

### QTL analysis of awn-related traits

CS showed a characteristic curved short awn (Fig 1A), with an awn length of 6.69 ± 0.75 mm at the top and 4.06 ± 0.55 mm at the middle of the spike. In contrast, the awns of M808 were relatively straight and short at the middle of the spike (5.05 ± 0.95 mm) but relatively long (30.45 ± 7.79 mm) at the top of the spike (Fig 1B). In a RIL population derived from CS and M808, the awn length at the middle of spike ranged from 0.88 to 81.93 mm with an average of 15.30 ± 21.35 mm, and the length ranged from 0.98 to 78.42 mm at the top of spike with an average of 18.05 ± 19.76 mm. Many of the RILs showed an awn length ≤ 5 mm (Fig 1H).

We performed QTL analysis for awn length using a high-resolution map constructed in our previous study [26] and identified three main QTLs for awn length at the top and middle of the spike located on chromosomes 4A, 5A and 6B (Table 1). The 4A QTL, corresponding to *Hd* locus, was located around 20 cM with a LOD score greater than 38 and explained over 23.1% of the phenotypic variation in the RIL population. The LOD score of the 5A QTL (the *B1* locus), located at the telomeric region of the long arm (306 cM), was higher than 47 and explained more than 30.1% of the phenotypic variance. The 6B QTL, corresponding to *B2* locus, was identified in the centromeric region with a LOD score of around 50, and around 30% of the phenotypic variation could be explained by this QTL. The CS allele at the 4A and 6B QTLs and the M808 allele at the 5A QTL decreased the awn length.

**Table 1:** Summary of the identified QTLs and epistasis.

Chr.	Position (cM)	LOD score	Closest marker	Estimated effect^a^	PV (%)	*P*-value^b^
**Awn length at the top of spike**						
4A	22.0	48.8	*Xgwm165_1*	10.69	31.2	< 2e-16
5A	306.0	47.8	*Xgwm291*	-9.16	30.1	< 2e-16
6B	131.5	47.2	*WABM213790*	10.31	29.5	< 2e-16
4A x 5A^c^	-	19.0	-	-4.65	8.3	< 2e-16
4A x 6B^c^	-	6.7	-	2.79	2.5	5.3e-08
5A x 6B^c^	-	5.9	-	-2.69	2.2	3.6e-07
**Awn length at the middle of spike**						
4A	20.0	38.5	*WABM242596*	8.75	23.1	< 2e-16
5A	306.0	61.2	*Xgwm291*	-13.20	50.0	< 2e-16
6B	131.5	50.5	*WABM213790*	11.32	35.5	< 2e-16
4A x 5A^c^	-	21.5	-	-6.00	10.4	< 2e-16
4A x 6B^c^	-	4.5	-	2.33	1.8	8.8e-06
5A x 6B^c^	-	26.0	-	-6.47	13.3	< 2e-16
**Hooded phenotype**						
4A	22.0	22.0	*Xgwm165_1*	-0.14	31.2	< 2e-16
5A	302.0	11.6	*WABM232824*	-0.07	14.6	3.7e-11
6B	123.1	10.5	*WABM118519*	-0.10	13.0	4.5e-10
4A x 5A^c^	-	6.4	-	0.09	7.5	1.1e-07
4A x 6B^c^	-	5.5	-	0.09	6.4	8.4e-07
5A x 6B^c^	-	2.0	-	0.06	2.2	3.2e-03

^a^Positive values indicate that M808-allele increase the trait value.

^b^*P*-value of the *F*-test indicating the goodness of fit of a given QTL or QTL x QTL interaction in the multiple QTL model.

^c^Epistasis between the QTLs.

**Fig 2:**
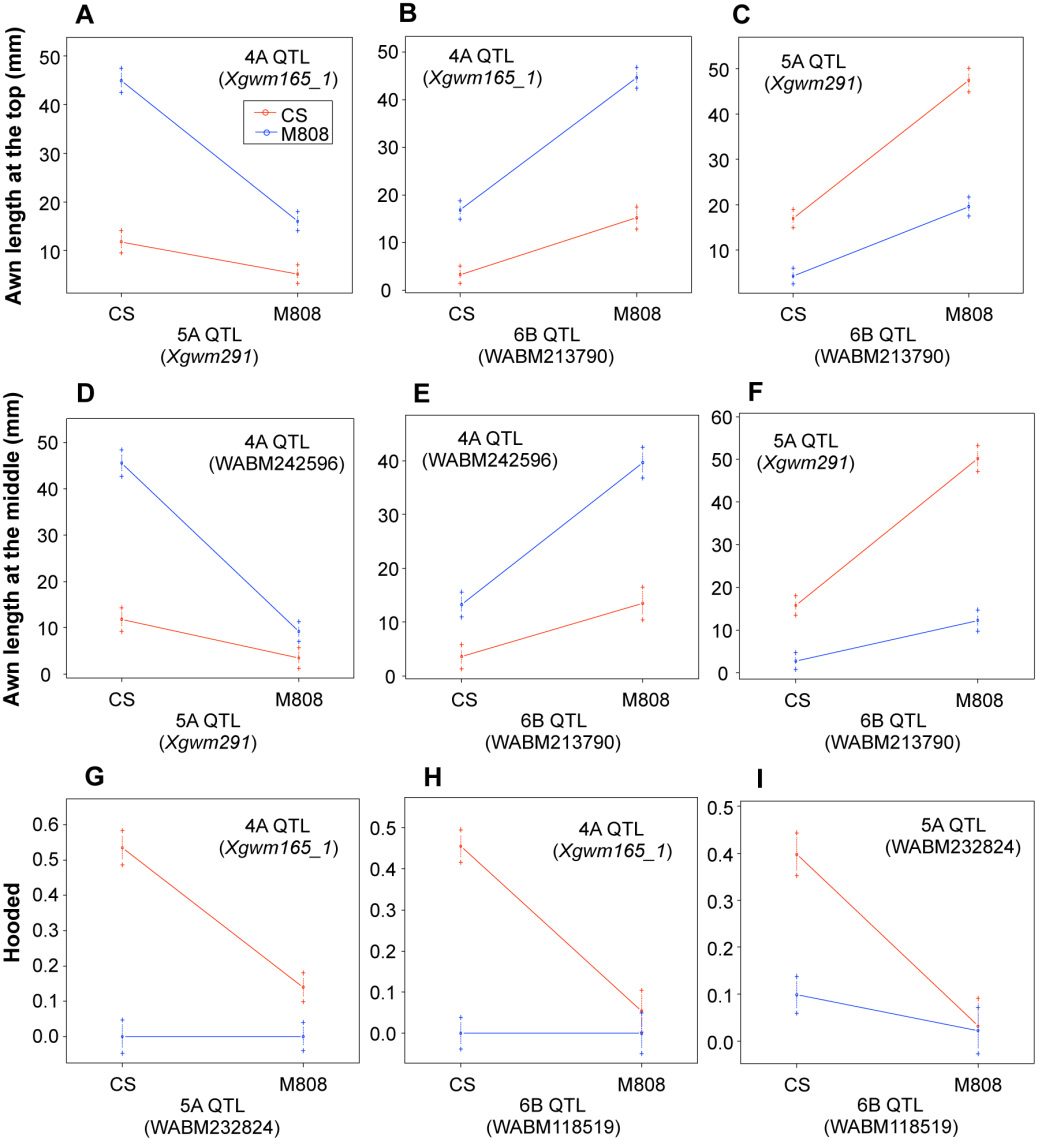
Two-way interaction plots for the three identified QTLs. Two-locus genotypic effects for awn length at the top (A to C) and middle (D to F) and for the hooded phenotype (G to I) were plotted using the genotype data of the markers with the highest LOD score. Red lines represent a genotype homozygous for CS and blue lines represent a genotype homozygous for M808. Error bars are ± standard errors.

Significant QTL x QTL interactions (epistasis) were also observed among these three loci (Table 1, Fig 2). Interaction between the 4A and 5A QTLs had the greatest effect on awn length at the top of the spike, with a LOD score of 19, and explained 8.3% of the phenotypic variation. 4A x 6B and 5A x 6B interactions exhibited a LOD score of 6.7 and 5.9, respectively, explaining 2.5 and 2.2% of the phenotypic variation. On the other hand, 4A x 5A and 5A x 6B interactions exhibited a great effect on awn length at the middle of the spike, with a LOD score higher than 21, and could explain more than 10% of the phenotypic variation.

**Fig 3:**
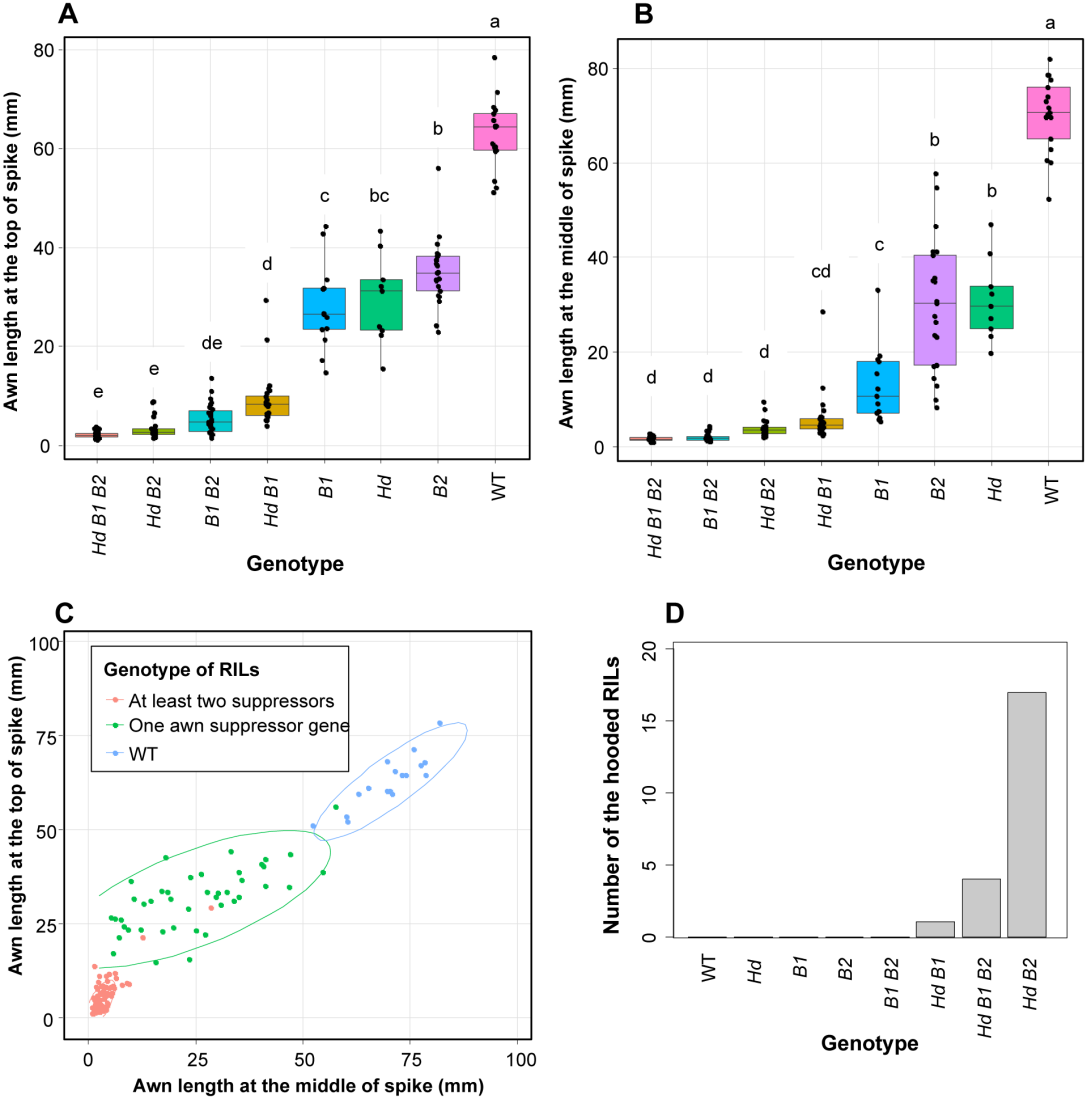
Comparison of awn length and hooded phenotype among different genotypes of RILs. Comparison of awn length at (A) the top and (B) the middle of the spike for the eight combinations of *Hd*, *B1*, *B2* and WT alleles in RILs. ANOVA with a *post-hoc* Tukey’s HSD test was performed and genotypes with the same letter did not present significant differences in awn length (*P* < 0.05). The phenotypic data of the parental lines (CS [*Hd B2*] and M808 [*B1*]) are indicated by red dots. (C) Scatter plot of awn length at the top (*y*-axis) with respect to that of the middle of the spike (*x*-axis). Three groups are observed: WT (blue dots), RILs with one of the dominant awning inhibitor alleles (green) and RILs with at least two dominant alleles (pink). Awn length of the parental lines are indicated by black dots. (D) Frequency distribution of RILs with hooded phenotype according to genotype.

Comparing the genotype data of flanking markers at the three QTLs with the phenotype data, we identified RILs with dominant *Hd* (CS allele at the 4A QTL), *B1* (M808 allele at the 5A QTL) and *B2* (CS allele at the 6B QTL) alleles and recessive wild type (WT) alleles (S3 Table). We found 17 RILs with WT alleles at the three loci (*hdhdb1b1b2b2*), nine RILs with only the dominant *Hd* allele (*HdHdb1b1b2b2*), 13 RILs with only the dominant *B1* allele (*hdhdB1B1b2b2*) and 21 with only the dominant *B2* allele (*hdhdb1b1B2B2*). In the case of RILs with two dominant awning inhibitor genes, 24 RILs had *Hd* and *B1*, 22 had *Hd* and *B2*, and 27 had *B1* and *B2* alleles. On the other hand, 28 RILs with all three awning inhibitor genes were found. There were no significant differences in awn length at the top of spike between RILs with two or three awning inhibitor genes, except for those containing *Hd* and *B1*, which had slightly longer awns (Fig 3A). RILs containing only one inhibitor gene exhibited intermediate awn length at the top compared with WT and lines containing at least two inhibitors. In contrast, no differences were observed in awn length at the middle of the spike among individuals with two or three awning inhibitors (Fig 3B), or between individuals with *HdB1* (*HdHdB1B1b2b2*) or *B1* (*hdhdB1B1b2b2*) genotypes. RILs with *Hd* (*HdHdb1b1b2b2*) and *B2* (*hdhdb1b1B2B2*) genotypes presented an intermediate awn length, as observed at the top of the spike. Based mainly on awn length at the top of the spike, the RILs can be grouped into WT, lines containing one inhibitor, and individuals with at least two inhibitors (Fig 3A and 3C).

In this RIL population, the hooded phenotype (Fig 1C to 1E) was also observed in some individuals (31 out of 210 RILs) but not in the parents. For QTL analysis, we assigned a trait value of “1” to the RILs with a membranous structure (Fig 1C and 1D) or broadening of the base of awn (Fig 1E), and “0” to RILs without a membranous structure or broadening because it was difficult to quantify this trait. QTL analysis revealed that the three QTLs for awn length are also involved in development of the hooded phenotype (Table 1). These loci explained 31.2 (4A QTL), 14.6 (5A QTL) and 13.0% (6B QTL) of the phenotypic variation. CS alleles at the three QTLs contributed to appearance of the hooded phenotype. Significant epistasis was also observed among these QTLs (Table 1, Fig 2G to 2I), for which the effects of 4A x 5A and 4A x 6B interactions were higher than 5A x 6B interaction. There were no differences in the allelic effects of the 5A and 6B QTLs when the genotype of 4A QTL was fixed to the M808 allele, and the hooded phenotype was not expressed under these conditions. Therefore, this phenotype might only be observed when an individual contains a CS allele at the 4A QTL, and also the 5A and 6B QTLs. Indeed, in the 161 RILs with known genotypes, 17 of the 22 hooded RILs contained homozygous *Hd* and *B2* alleles (Fig 3D), and formed a membranous outgrowth (Fig 1C and 1D). The remaining five hooded RILs presented *HdHdB1B1b2b2* or *HdHdB1B1B2B2* genotypes and only a broadening of the base of awns was observed, without formation of a membranous structure (Fig 1E). In a near-isogenic line (NIL) (*HdHdb1b1b2b2*) of common wheat cv. S-615 (*hdhdb1b1b2b2*) with *Hd* from CS (named *Hd*-S615), broadening of the base of awns could be observed (Fig 1F) and sometimes a membranous structure was also formed without the presence of *B2* locus (Fig 1G).

### Location of candidate genes on wheat genome

To test whether the genes known to be involved in awn development in rice and barley could be causal genes of the *Hd*, *B1* and *B2* loci, we performed a BlastP search against wheat protein sequences of the EnsemblPlants *Triticum aestivum* database (http://plants.ensembl.org/Triticum_aestivum/Info/Index). An ortholog of *DL* was located on chromosome 4AS and an ortholog of *RAE2* on chromosome 6BL (S4 Table). To check their location, contig sequences of the array-based markers were mapped on wheat genomic scaffolds and then the genes contained in these scaffolds were searched against proteins in the rice (http://rapdb.dna.affrc.go.jp/), barley (http://plants.ensembl.org/Hordeum_vulgare/Info/Index), or both databases using the Blast algorithm. Based on the synteny between wheat and the barley and rice genomes, the locations of these two genes were predicted on the wheat genome.

**Fig 4:**
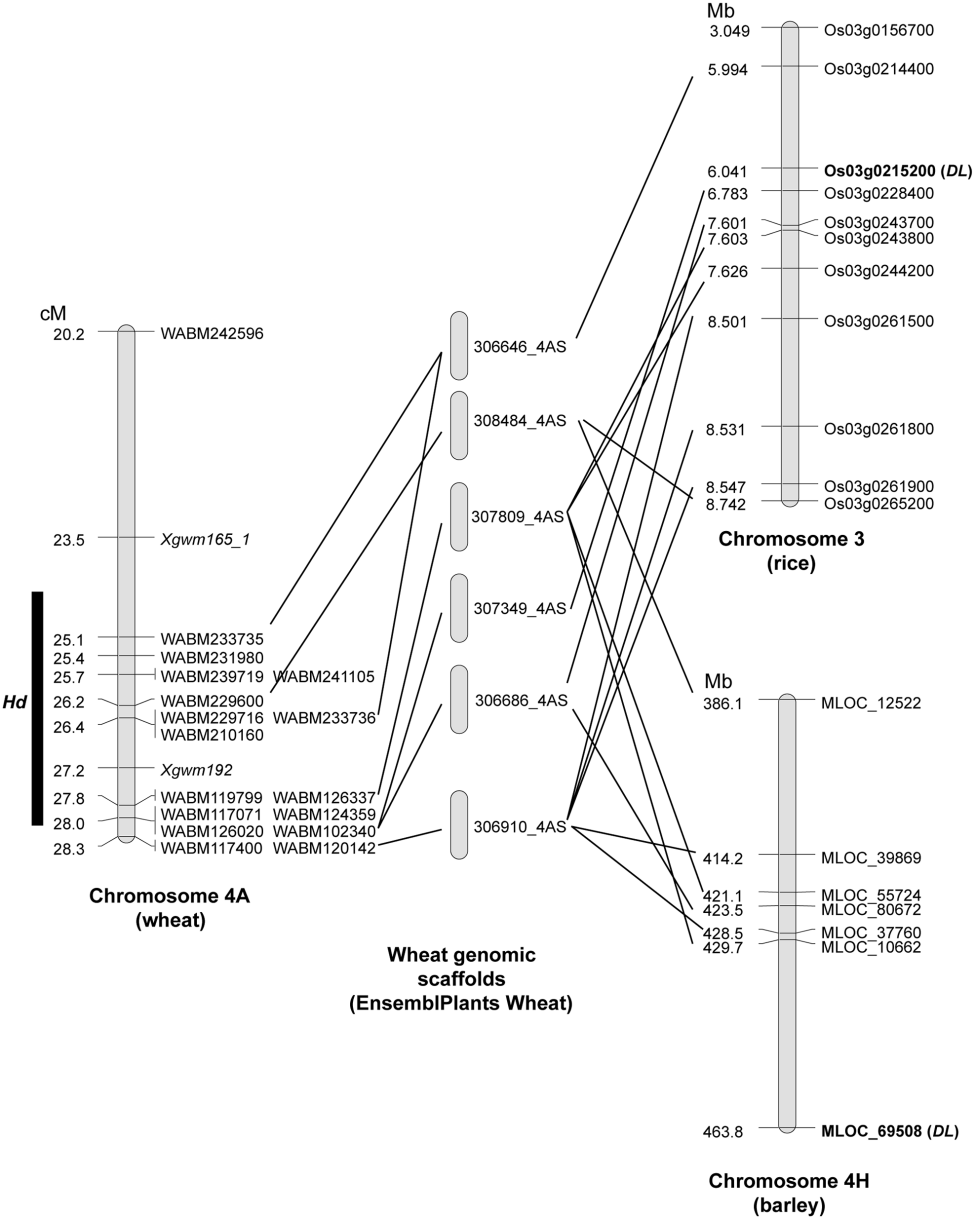
Chromosomal synteny of *Hd* and orthologous regions among wheat, rice and barley. Using the sequence of markers located around the *Hd* locus (left map), a Blast search was performed to find the corresponding genomic scaffolds (middle panel). Genes present on each scaffold were used to search for orthologs in rice and barley. Comparing our genetic map of wheat with the physical map of rice chromosome 3 (top of right panel), we located *DL* near the *Hd* locus. On the other hand, this region of chromosome 4A is known to have an inversion with respect to the corresponding region of chromosome 4H (bottom of right panel). Therefore, the ortholog of *DL* in barley might be around the *Hd* locus, but the exact location could not be determined.

**Fig 5:**
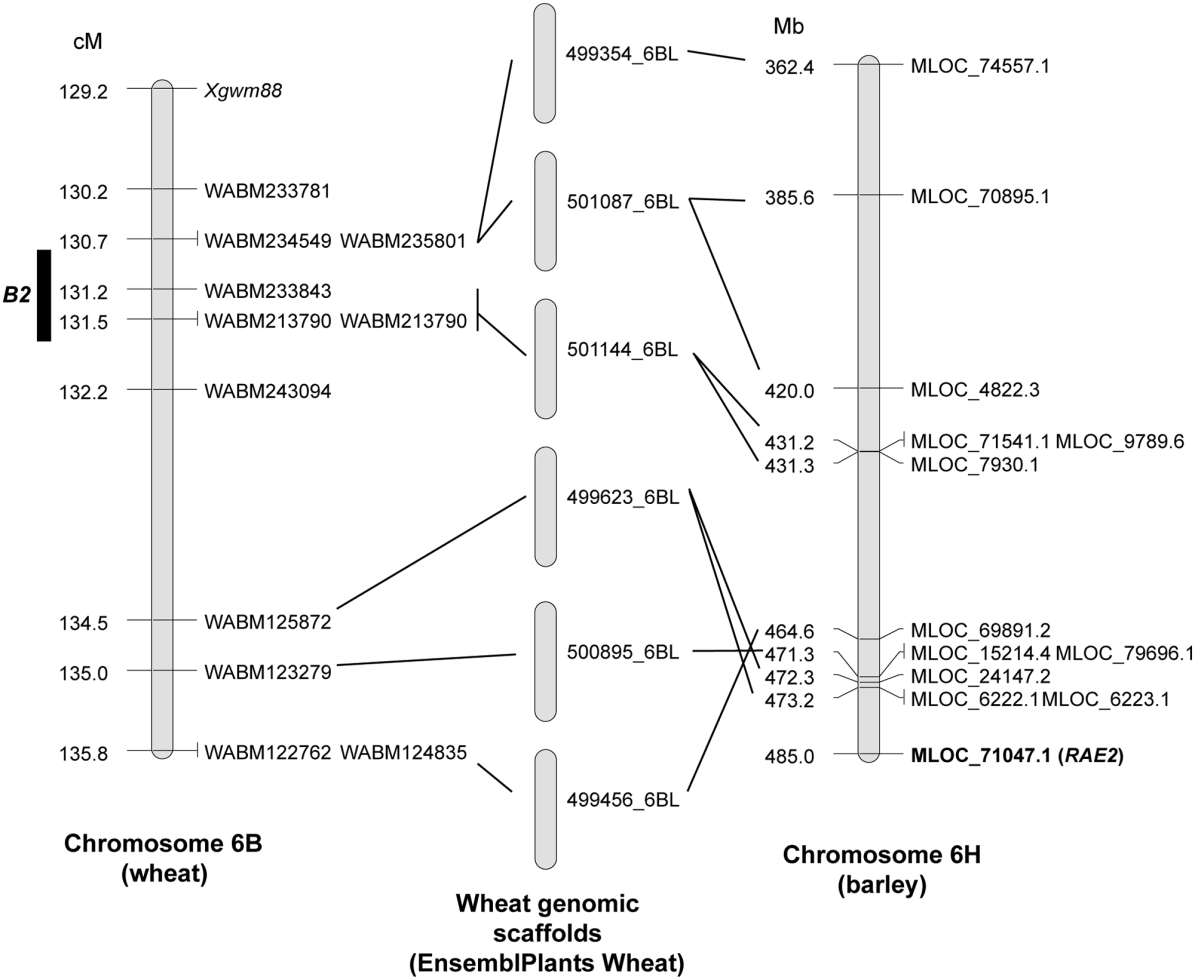
Chromosomal synteny between wheat chromosome 6B and barley chromosome 6H. The region around the *B2* locus was compared with the corresponding region of the barley genome to predict the location of the *RAE2* gene (MLOC_71047.1). The left map (genetic map) corresponds to chromosome 6B of wheat, the middle panel shows the genomic scaffolds of wheat containing the indicated markers and the right map (physical map) is barley chromosome 6H, where the orthologs of the genes on the wheat scaffolds are indicated.

Nine rice genes were found as orthologs of the wheat genes contained in the genomic scaffolds, and *DL* seemed to be located around the *Hd* locus (Fig 4). Because only six barley orthologs were found, it was difficult to compare this genomic region between barley and wheat. Alignment of the *DL* homologs indicated that this gene on chromosome 4A is functional and that the differences in the protein sequence were no greater than for other wheat genomes and other species (S2 Fig). Similarly, the *RAE2* location was predicted based on the synteny with barley using 12 orthologs (Fig 5). This result indicated that *RAE2* was near but not at the *B2* locus. Wheat and barley group 6 chromosomes are syntenic to rice chromosome 2 [35,36], but the ortholog of *RAE2* (located on chromosome 8 of rice) in barley and wheat was located on the long arm of group 6 chromosome, distal to the *B2* locus. We did not compare the results for rice because no orthologs of rice chromosome 8 were found in this *B2* region.

### Variation of awn length in the hexaploid wheat core collection

**Fig 6:**
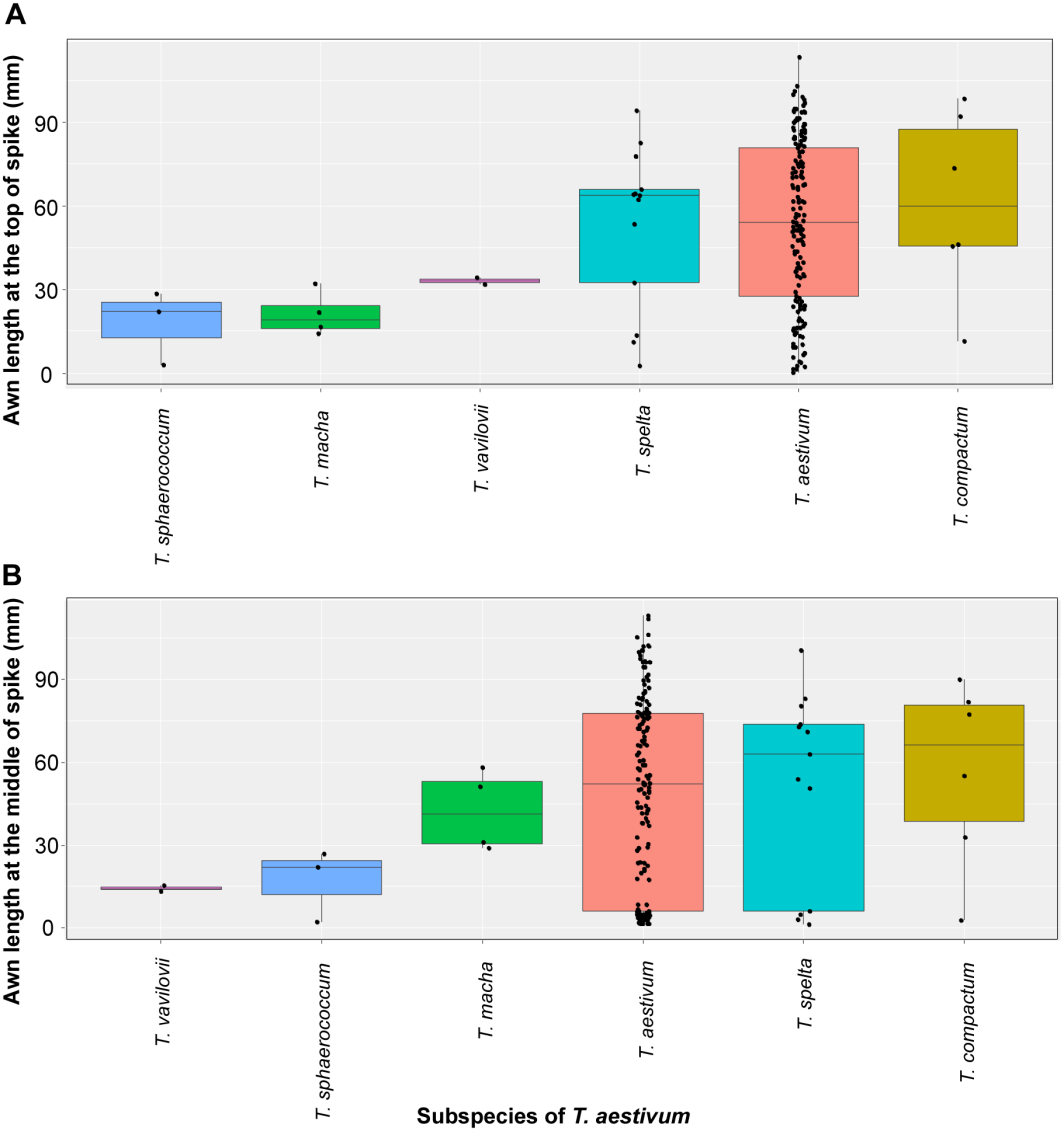
Box and whisker plots for comparison of awn length at the top (A) and middle (B) of the spike in the six subspecies of *T*. *aestivum*.

A hexaploid wheat core collection, established by NBRP-Wheat [28], included 161 common wheat (*T*. *aestivum* subsp. *aestivum*) varieties and the following subspecies of *T*. *aestivum*: *compactum* (*n* = 6), *macha* (*n* = 4), *spelta* (*n* = 13), *sphaerococcum* (*n* = 3) and *vavilovii* (*n* = 2), which were collected worldwide (S1 Table). The awn length at the top of the spike ranged from 0.16 to 113.27 mm (S3A Fig), with an average of 52.88 ± 29.31 mm, and at the middle of the spike, from 1.24 to 112.99 mm, with an average of 48.22 ± 33.97 mm (S3B Fig). Of the six subspecies analyzed, *aestivum*, *compactum* and *spelta* showed wide variation in awn length (Fig 6). In contrast, *macha*, *sphaerococcum* and *vavilovii* exhibited shorter awns than other subspecies. In *vavilovii*, awn length at the top was longer than at the middle of the spike. On the other hand, awns at the middle of the spike were longer in *macha*. Hexaploid wheat varieties from Jordan, the USA, Iraq, India, Greece, Spain, Iran, Romania, Mexico and Egypt mostly presented a long awn at the top and middle of the spike (S4 Fig), and varieties from Bhutan, Italy, the UK, Australia and Syria presented short awns throughout the spike. The awn length of wheat varieties from Canada and Lebanon were short at the middle but long at the top of the spike. In contrast, varieties from Georgia presented relatively short awns at the top but long awns at the middle of the spike.

### Conversion of array-based markers to PCR-based markers

To determine the genotype of the NBRP wheat core collection at the three QTLs, we attempted to convert the array-based markers to PCR-based ones. First, we selected 40 array-based markers around the peak of each QTL (21 markers from chromosome 4A, seven from 5A and 12 from 6B). Using the read or contig sequence of each marker, we performed a BlastN search against the hexaploid wheat genomic survey sequence (S1 Fig). No hits were found for two reads/contigs from chromosome 5A and one from 6B. Because the array-based markers were developed using genomic fragments with *Pst*I sites at both ends and without any *Bst*NI sites within the fragment [26], putative *Pst*I sites were searched at both ends of the alignment of the read/contig sequence and genomic scaffold. CAPS markers were developed using the genomic scaffold with *Pst*I sites. If *Pst*I restriction sites were not found, SSR motifs were searched for in the genomic scaffold to develop SSR markers when the read or contig was derived from CS. For sequences derived from M808, we searched for SNP, indel and SSR motifs to develop PCR-based markers. Finally, we checked for polymorphisms in 31 of the markers developed (12 from chromosome 4A, two from 5A and 17 from 6B) of which 12 (four from chromosome 4A, one from 5A and seven from 6B) allowed detection of polymorphisms between CS and M808. Using these markers, RILs derived from CS and M808 were genotyped and genetic maps were constructed to confirm their locations. All 12 markers were located near the awning inhibitor loci (S5 Fig).

### Prediction of *Hd*, *B1* and *B2* alleles in the wheat core collection

**Fig 7:**
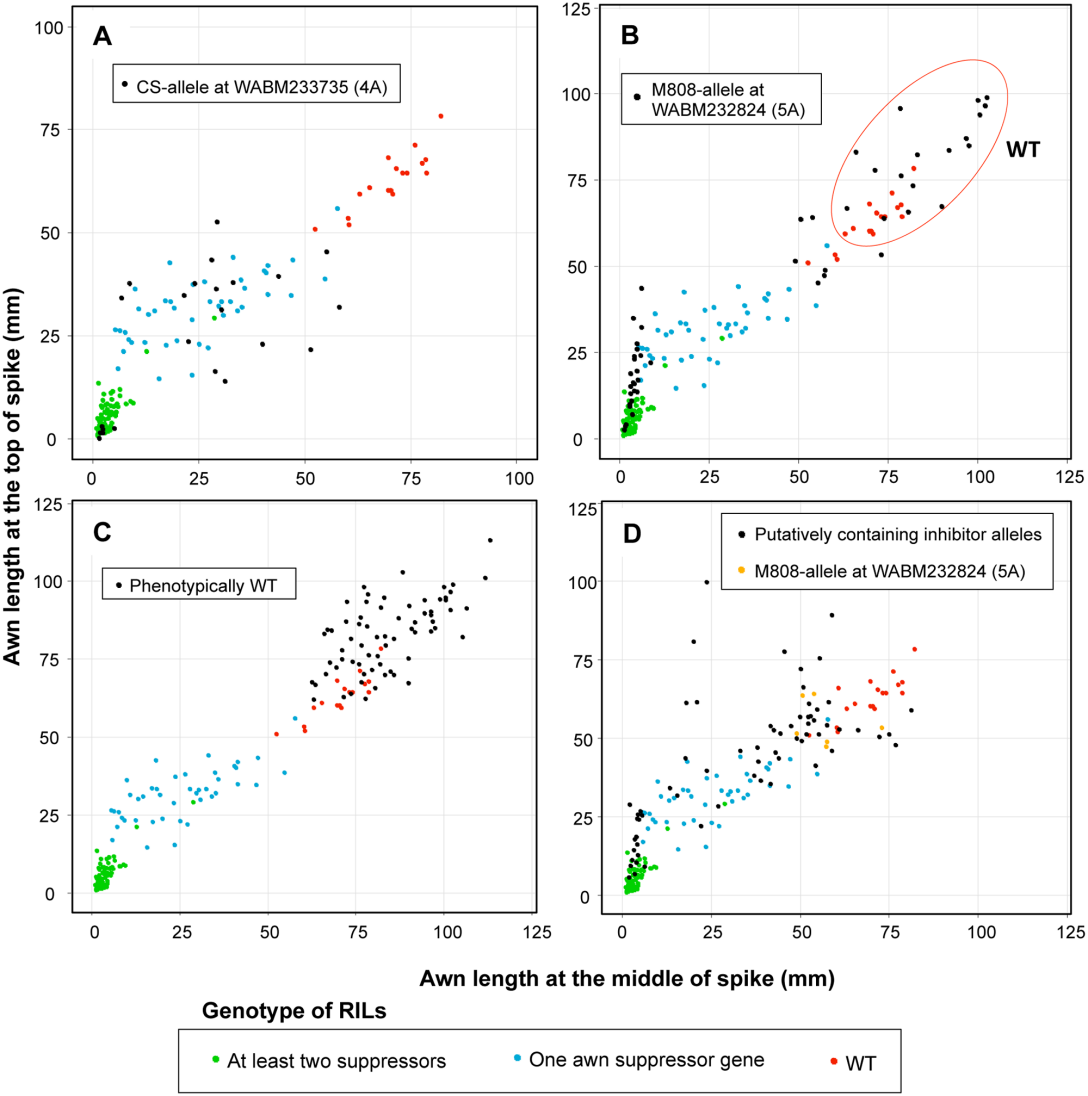
Comparison of phenotype of RILs with known genotype and hexaploids with predicted genotypes. The awn length of hexaploids with a putative *Hd* allele (A) and *B1* (B) allele was compared with that of RILs with known genotypes. WT RILs are indicated by red dots, those with one dominant awning inhibitor by blue, RILs with at least two inhibitors by green and hexaploid wheat lines by black. Individuals with an awn length at the top and middle of the spike > 60 mm (red circles) were considered WT. The awn length of phenotypically WT hexaploids (C) and lines putatively containing inhibitor alleles (D) was also compared with the RILs. Hexaploids with the M808-allele at *WABM232824* (near the *B1* locus) are indicated by orange dots.

Using the 12 markers developed and two publicly available SSR markers (*gwm192* on chromosome 4A and *gwm291* on 5A), 189 hexaploid wheat lines of the NBRP core collection were genotyped (S5 Table). The SSR markers *gwm291* and *WABM229716* were discarded because of the presence of multiple alleles, and the band pattern was similar to CS or M808 in only a few accessions. Based on the identified genotypes, the average awn length was compared between varieties with the CS and M808 alleles. Awn length was significantly lower in varieties with the CS allele at the markers for chromosome 4A, except for *WABM241105*, for which the opposite effect was observed (S6 Table). Because a greater difference in awn length was associated with marker *WABM233735*, individuals with the CS allele at this marker were selected and their phenotype was compared with that of 161 RILs with known genotypes at the three awning inhibitor loci. The phenotype of 23 wheat varieties with the CS allele coincided with RILs containing at least one awning inhibitor gene, but not with that of WT (Fig 7A). Of the 23 varieties, six seem to have at least one additional inhibitor of awn development. The 23 hexaploids were mainly composed of Asian wheat varieties (Table 2), and the subspecies *aestivum* was the most abundant, with 17 varieties, followed by all four *macha* varieties, one of *sphaerococcum* and one of *compactum* (Table 3).

**Table 2:** Distribution of some varieties with probable *Hd* and *B1* alleles and WT by country.

Country	Total	Probable *Hd*	Probable *B1*	WT	Unknown
Afghanistan	15	1	0	9	5
Australia	2	0	2	0	0
Bhutan	4	3	0	0	1
Canada	1	0	1	0	0
China	16	7	0	1	8
DDR	8	0	3	3	2
Ethiopia	9	1	1	4	3
Georgia, USSR	3	1	0	0	0
Greece	5	0	0	4	1
Iran	19	3	0	16	0
Japan	35	0	3	4	28
Lebanon	1	0	1	0	0
Nepal	6	3	1	0	2
Pakistan	7	0	0	4	3
Romania	5	0	1	4	0
Spain	8	0	0	5	3
Syria	1	0	1	0	0
Turkey	12	1	3	3	5
UK	6	0	5	1	0
USA	5	0	0	5	0
USSR	7	0	0	2	5
Others	14	1	3	5	5

**Table 3:** Distribution of probable *Hd* and *B1* dominant alleles in the subspecies of *T*. *aestivum*.

Subspecies of *T*. *aestivum*	Total	Probable *Hd*	Probable *B1*	WT	Unknown
ssp. *aestivum*	161	17	21	61	62
ssp. *spelta*	13	0	4	6	3
ssp. *compactum*	6	1	0	3	2
ssp. *macha*	4	4	0	0	0
ssp. *sphaerococcum*	3	1	0	0	2
ssp. *vavilovii*	2	0	0	0	2

For markers linked to the *B1* locus, only one could be used for genotyping of the wheat core collection, and a significant difference in awn length at the top of the spike was observed between lines with the CS and M808 alleles (S6 Table). Out of 49 wheat varieties, 24 with the M808 allele at marker *WABM232824* presented a phenotype similar to WT (Fig 7B). We considered these lines as WT if the awn length at both the top and middle was greater than 60 mm based on the observation that a RIL with the *B2* genotype had a respective length of 56.04 mm and 57.73 mm at the top and middle of the spike (the longest among the non-WT varieties). Six varieties with a WT-like phenotype but with a length less than 60 mm were considered to have an unknown genotype. Of the remaining 45 varieties, six seem to have at least one additional gene inhibiting awn development. The dominant *B1* allele seems to be present mainly in hexaploid varieties from the UK and other countries such as Australia, Germany, Japan and Turkey (Table 2), and was found only in the subspecies *aestivum* and *spelta* (Table 3). Although one hexaploid presented the CS allele at *WABM233735* of chromosome 4A and the M808 allele at *WABM232824*, the awn length was 45.43 mm at the top and 55.18 mm at the middle of the spike, a phenotype longer than expected.

In one marker linked to *B2* on chromosome 6B (*WABM125872*) awn length at the top of the spike was significantly higher (*P* = 0.015) in individuals with the CS allele, which was the opposite of the expected effect (S6 Table). Thus, we were not able to predict the presence of the *B2* dominant allele in the NBRP wheat core collection. Based on the criterion mentioned above, 61 varieties belonging to subspecies *aestivum*, six to *spelta* and three to *compactum* were classified as WT (Table 2, Fig 7C). Most of the hexaploid varieties from the USA, Iran, Spain and Romania exhibited an awn length similar to the WT of the RIL population (Table 3). In total, the allele composition of 71 varieties with short awns could not be explained using the markers analyzed, including most of subspecies *sphaerococcum* and *vavilovii* (Table 3, Fig 7D). Most of the hexaploid varieties from Japan and USSR remained categorized as as unknown genotype.

## Discussion

Although important roles of awns in spike photosynthetic activity and seed dispersal have been suggested in wheat, the genes involved in awn development have not been identified. In this study, the dominant inhibitors of awn development, *Hd*, *B1* and *B2*, were mapped to a high-density genetic map of a RIL population derived from CS and M808 [26], which indicated that the genotypes of CS and M808 are respectively *HdHdb1b1B2B2* and *hdhdB1B1b2b2*. Sourdille et al. [21] suggested that the genotype of CS is *HdHdB1B1B2B2* based on the observation that the CS deletion line 5AL-10 was slightly awned (bearded). This deletion line lacks the telomeric region of the long arm of chromosome 5A, where the break point is located between two SSR loci, *Xgwm156* and *Xgwm617* [37] (S6 Fig). However another CS deletion line, 5AL-17 (which has the break point between *Xcfa2163* and *Xcfa2155*), showed an awnless phenotype [21]. If CS contains the *B1* allele, these earlier results indicate that the *B1* locus should be located between the break point of 5AL-10 and 5AL-17 (between the two SSR loci, *Xgwm156* and *Xcfa2155*). We found that *B1* is located distal to the break point of 5AL-23, near the telomere, as previously reported [38]. Our results indicate that CS has the *b1* homozygous allele, as previously reported [39].

QTL analysis for awn length indicates that the *Hd* locus has a large effect at the top of the spike and the *B1* locus at the middle of the spike. Significant genetic interactions among the three awning inhibitors were also observed, mainly between *Hd* and *B1* for awn length at the top of the spike and between *B1* and other two loci at the central part of the spike. These observations imply that the awn length of individuals with two or more inhibitors cannot be explained only by the additive effect of each dominant allele, and that the combination of awning inhibitor alleles at different loci potentiates the suppression of awn elongation. A stable awnless phenotype can only be observed when all three dominant alleles are present (Fig 3). Because the linkage map used in this study was constructed using array-based markers, we selected markers tightly linked to the three loci and attempted to convert them into PCR-based markers. Using these PCR-based markers, 189 hexaploid wheat varieties with a wide variation in awn length were genotyped. A significant association was observed between the genotype of the marker *WABM233735* (tightly linked to *Hd*) and the phenotype (S5 Fig, S6 Table). The phenotype of hexaploid wheat varieties with the CS allele overlapped the phenotype of RILs with at least one awning inhibitor (Fig 7A). Many of these hexaploids were cultivated in Asia (Table 2), and this is consistent with the report that some Chinese varieties present curved awns with a reduced length (classified as hooded bearded) or very short hook-shaped awns (hooded beardless) [18].

Although *WABM232824* was not necessarily tightly linked to *B1*, hexaploids with the M808 allele at this marker tended to have shorter awns than those with the CS allele. Around 50% of these wheat lines with the M808 allele presented a WT-like phenotype (Fig 7B), indicating that there is no strong linkage disequilibrium between *WABM232824* and *B1*. In a previous study, association between the *B1* locus and a short awn phenotype was observed in a panel of 64 wheat varieties of predominantly UK origin [25], which is consistent with our prediction that all of the UK varieties with short awns contained the *B1* allele. Many markers tightly linked to *B2* were also found on the linkage map. However, we could not observe any significant association between phenotype and genotype in the hexaploid wheat core collection. Because *B2* is located near the centromeric region, where the recombination rate is low [40], many markers appeared to be closely linked to *B2* in our mapping population. In a population derived from a biparental cross, QTL mapping is performed based on recent recombination events. However, historic recombination is used to assess phenotype-genotype correlation in a natural population. Therefore, recombination events might have occurred between *B2* and the *B2*-linked markers analyzed in the hexaploid core collection.

At the subspecies level, many *T*. *aestivum* subsp. *aestivum* varieties were found to have *B1* or *Hd* alleles. However, 62 of the 100 varieties with short awns remained classified as of unknown genotype. In the subspecies *spelta*, around half of the lines with short awns were predicted to contain the *B1* allele. However, we could not determine the genotype of *vavilovii* accessions or of many accessions of *sphaerococcum* and *compactum*. Other genes may be involved in the reduced awn length in the hexaploids with unknown genotype. In addition, there are no reports on *Hd* alleles in *compactum*, *macha* or *sphaerococcum*. This indicates that the marker *WABM233735* is not tightly linked to *Hd*. Therefore, markers that represent strong linkage disequilibrium to the *Hd*, *B1* and *B2* loci should be developed to precisely determine the genotypes of comprehensive collections of hexaploid wheat accessions.

**Fig 8:**
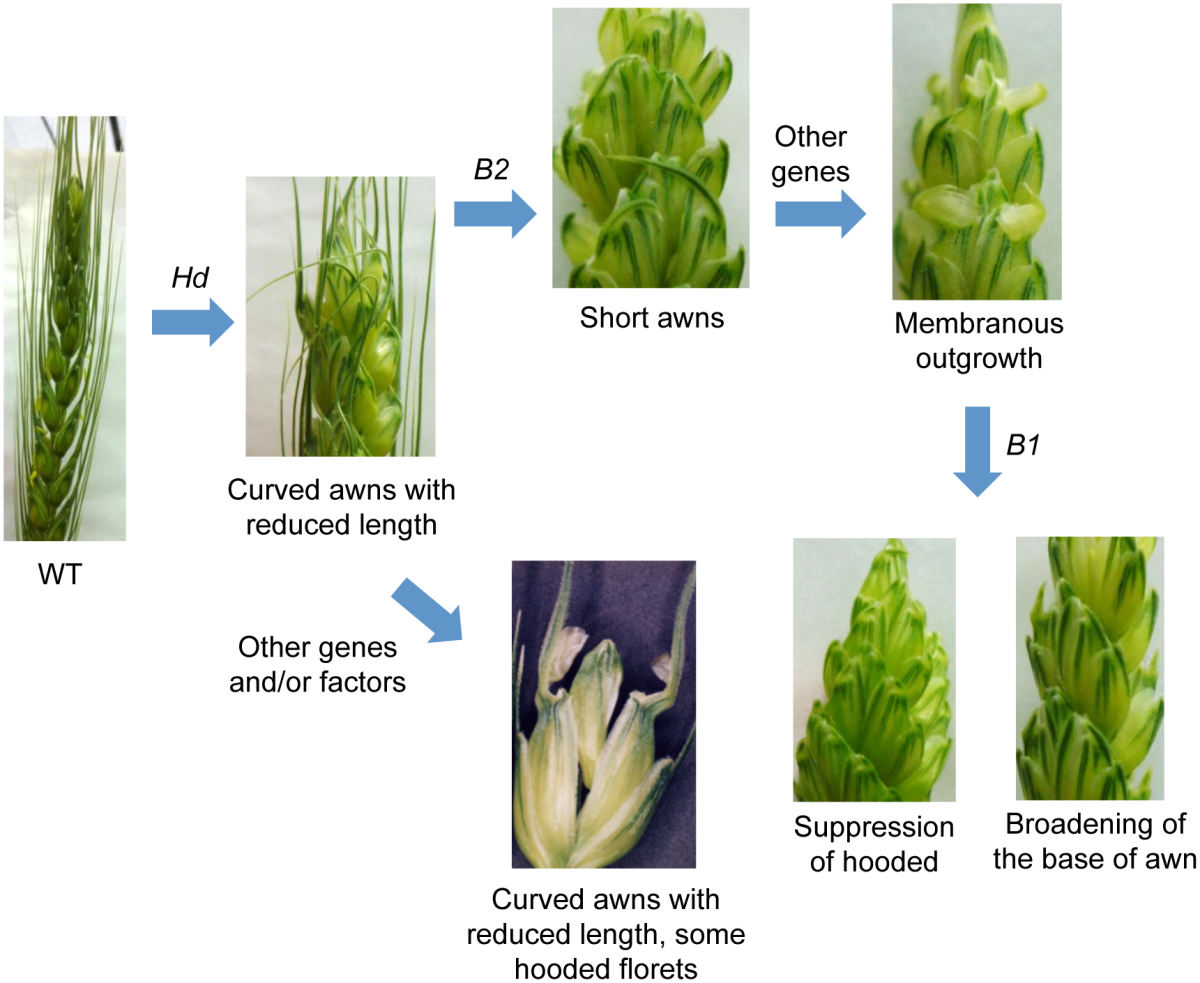
Model of interactions among *Hd*, *B1* and *B2* for development of the hooded phenotype. The dominant *Hd* allele is necessary for development of the membranous lateral expansion at the base of awns. Presence of the dominant *B2* allele and other factors contribute to the formation of this ectopic tissue. In contrast, presence of the dominant *B1* allele suppresses its formation, leading to a complete lack of the hooded phenotype or to a broadening of the base of awn, depending probably on the genetic background.

In our mapping population, RILs with the hooded phenotype were also observed. We found that in addition to the *Hd* locus, *B1* and *B2* are involved in the development of the hooded phenotype. The dominant *B2* allele seems to be important for the formation of the membranous outgrowth at the base of the awn in individuals with the dominant *Hd* allele (Figs 2H and 3). Although CS contains the *Hd* and *B2* alleles, it did not develop this phenotype, indicating the involvement of another locus or loci with M808 allele in the RIL population that could not be identified in our QTL analysis. In contrast, in the NIL of S-615 with the *Hd* allele (*HdHdb1b1b2b2* genotype), the membranous structure was also observed in some cases (Fig 1G). Moreover, Watkins and Ellerton [18] stated that hoodedness is considerably exaggerated in late tillers. These observations suggest that *Hd* is essential for expression of hoodedness, and that other genetic factors (such as *B2*) and developmental stage-dependent factors potentiate the stable development of this membranous structure (Fig 8). To identify these genetic factors, the hooded phenotype must be better quantified, since we only used trait values of “1” and “0” for QTL analysis. The hooded phenotype, in *sensu stricto*, is the formation of a membranous lateral expansion, but we also considered the broadening of the base of the awn as a hooded trait based on our hypothesis that this is an intermediate phenotype of hooded. However, other intermediate phenotypes or different degrees of phenotype expression might exist. On the other hand, the dominant *B1* allele seems to act as a suppressor of membranous outgrowth formation (Figs 2G and 3), and only a broadening of the base sometimes occurs when the *Hd* allele is present. This relationship is similar to *Kap* (*Hooded*) and *Lks2* or *suK* (suppressor of *Kap*) in barley, where *Lks2* and *suK* act as suppressors of the *Hooded* phenotype [41,42].

Several genes involved in awn development have been reported in rice and barley [11–15, 17]. Although *DL* and *RAE2* are respectively located near the *Hd* and *B2* loci, neither seems to be a causal gene in wheat. In barley, the *Hooded* mutation leads to ectopic expression of *HvKnox3* at the tip of the lemma, producing an extra floret instead of an awn [17]. However, its ortholog in wheat, *Wknox1a*, is located on the long arm of chromosome 4A (S4 Table; [43]), not on the short arm, where the *Hd* locus is located. Although ectopic expression of *Wknox1* was confirmed in the lemma of the *Hd* NIL of S-615 [43], no structural mutation related to the hooded phenotype has been identified at the *Wknox1a* locus of CS and no functional or transcriptional differences were found among the three *Wknox1* homoeologs of CS [44]. Thus, *Wknox1a* on chromosome 4A appears not to be the causal gene of *Hd*.

These observations indicate that the awning inhibitors in wheat are not orthologs of known genes involved in awn development. Further study is needed to identify the causal genes. Using a high-resolution genetic map, we found markers tightly linked to the three main genes involved in inhibition of awn development in common wheat. Although there was incomplete linkage between these markers and the awning inhibitor genes, we found a significant correlation between genotype and phenotype at the *Hd* and *B1* loci in the hexaploid wheat core collection. We also found that the dominant *Hd* and *B2* alleles are also involved in the stable development of membranous structures at the base of awns and that the *B1* allele can suppress the hooded phenotype. The cloning of these genes might clarify the molecular mechanisms of awn formation and elongation and the development of the hooded phenotype in wheat.

## Supporting information

S1 Fig Schematic of conversion of array-based markers to PCR-based markers. First, BlastN searches of reads/contig sequences of the markers were performed against the wheat survey genome sequence. The aligned sequences were searched for *Pst*I restriction sites for development of CAPS markers. If no *Pst*I sites were found, SSR motifs were searched and primers were designed to amplify these motifs. If no SSR motifs were present in the genomic sequence and if the read/contig sequence was derived from M808, SNPs and indels were searched because the genome sequence was derived from CS. In total, 12 markers were developed and used for further analyses.
(PDF)

S2 Fig Alignment of DL and related protein sequences. Orthologs of rice DL (accession: Q76EJ0, OsDL) in *Brachypodium distachyon* (XP_010228969.1, BdDL), barley (BAK04901.1, HvDL), and the A-genome (306676_AA1011910.1, TaDL-A), B-genome (321049_AA1054410.1, TaDL-B) and D-genome of wheat (343031_AA1127860.1, TaDL-D) were aligned. Conserved amino acid residues are indicated by asterisks and the YABBY domain by the orange bar.
(PDF)

S3 Fig Variation in awn length of the 189 hexaploid wheat varieties. Awn length at the top (A) and middle (B) of the spike in the hexaploid wheat core collection. Subspecies of *T*. *aestivum* are indicated by different colors. Data are means ± SD.
(PDF)

S4 Fig Awn length of the hexaploids according to geographical origin. Box and whisker plots for awn length at the top (A) and middle (B) of the spike in the hexaploid wheat lines are grouped by geographical origin.
(PDF)

S5 Fig Linkage map constructed using PCR-based markers. The linkage map was constructed using 161 RILs with known genotypes at the *Hd*, *B1* and *B2* loci to confirm the locations of the PCR-based markers developed. SSR markers were also included.
(PDF)

S6 Fig Comparative map indicating the location of the *B1* locus. The physical map of chromosome 5A (left) and genetic maps (middle and right) were compared to locate the *B1* locus on the physical map. Arrows indicate the break point of the chromosome deletion lines obtained by Endo and Gill. SSR markers mapped to the physical map of chromosome 5A were used to locate the *B1* locus.
(PDF)

S1 Table The 189 hexaploid wheat varieties used in this study. (XLSX)

S2 Table Primers used in this study. *These two SSR markers have been previously described (http://wheat.pw.usda.gov/GG3/).
(PDF)

S3 Table Identification of RILs with the dominant *Hd*, *B1* and *B2* alleles at the three QTLs. The presence of the *Hd*, *B1* and *B2* alleles were estimated based on the genotypes of markers at each QTL. Alleles are indicated in yellow when the genotype and phenotype were consistent, and green indicates an allele estimated by phenotype. The LOD peak of QTLs for awn length (top and middle) was observed in markers indicated by red. The first to third columns indicate the chromosome, marker name and position in cM of each marker, respectively.
(XLSX)

S4 Table Orthologs of genes involved in awn development in wheat. A BlastP search was performed against the EnsemblPlants *Triticum aestivum* protein database.
(PDF)

S5 Table Genotype of the 189 hexaploid wheat lines according to the PCR-based markers. A: CS allele, B: M808 allele, -: missing or other allele
(XLSX)

S6 Table Comparison of awn length in hexaploid wheat collection based on the genotype of markers analyzed. (XLSX)

## References

[1] Dahlgren R, Clifford HT, Yeo PF. The families of the monocotyledons: Structure, evolution and taxonomy. New York: Springer; 1985.

[2] Hua L, Wang DR, Tan L, Fu Y, Liu F, Xiao L, et al *LABA1*, a domestication gene associated with long, barbed awns in wild rice. Plant Cell 2015; 27: 1875–1888. 10.1105/tpc.15.00260 26082172 PMC4531357

[3] Sorensen AE. Seed dispersal by adhesion. Annu Rev Ecol Syst. 1986; 17: 443–463.

[4] Elbaum R, Zaltzman L, Burgert I, Fratzl P. The role of wheat awns in the seed dispersal unit. Science 2007; 316: 884–886. 10.1126/science.1140097 17495170

[5] McDonough WT, Gauch HG. The contribution of the awns to the development of the kernels of bearded wheat. Maryland Agric Exp Stn Bull. 1959; 103: 1–16.

[6] Perlitius L. Der Einfluss der Begrannung auf die Wasserverdunstung der Ahren und die Kornqualität. Inaug Diss Univ Breslau; 1903.

[7] Evans LT, Bingham J, Jackson P, Sutherland J. Effect of awns and drought on the supply of photosynthate and its distribution within wheat ears. Ann Appl Biol. 1972; 70: 67–76.

[8] Evans LT, Rawson HM. Photosynthesis and respiration by the flag leaf and components of the ear during grain development in wheat. Aust J Biol Sci. 1970; 23: 245–254.

[9] Vervelde GJ. The agricultural value of awns in cereals. Neth J Agric Sci. 1953; 1: 2–10.

[10] Takahashi N, Alwan H, Alterfa H, Sato T. Significant role of awn in rice plants (1): A survey of agricultural value of rice awn. Rep Inst Agr Res Tohoku Univ 1986; 35: 21–31.

[11] Luo J, Liu H, Zhou T, Gu B, Huang X, Shangguan Y, et al *An-1* encodes a basic helix-loop-helix protein that regulates awn development, grain size, and grain number in rice. Plant Cell 2013; 25: 3360–3376. 10.1105/tpc.113.113589 24076974 PMC3809537

[12] Toriba T, Hirano H. The *DROOPING LEAF* and *OsETTIN2* genes promote awn development in rice. Plant J. 2014; 77: 616–626. 10.1111/tpj.12411 24330191

[13] Hua L, Wang DR, Tan L, Fu Y, Liu F, Xiao L, et al *LABA1*, a domestication gene associated with long, barbed awns in wild rice. Plant Cell 2015; 27: 1875–1888. 10.1105/tpc.15.00260 26082172 PMC4531357

[14] Bessho-Uehara K, Wang DR, Furuta T, Minami A, Nagai K, Gamuyao R, et al Loss of function at *RAE2*, a previously unidentified EPFL, is required for awnlessness in cultivated Asian rice. Proc Natl Acad Sci USA. 2016; 113: 8969–8974. 10.1073/pnas.1604849113 27466405 PMC4987784

[15] Yuo T, Yamashita Y, Kanamori H, Matsumoto T, Lundqvist U, Sato K, et al *A SHORT INTERNODES (SHI)* family transcription factor gene regulates awn elongation and pistil morphology in barley. J Exp Bot. 2012; 63: 5223–5232. 10.1093/jxb/ers182 22791834 PMC3430995

[16] Stebbins GL, Yagil E. The morphogenetic effects of the hooded gene in barley. I. The course of development in hooded and awned genotypes. Genetics 1966; 54: 727–741. 17248328 10.1093/genetics/54.3.727 PMC1211196

[17] Müller K, Romano N, Gerstner O, Garcia-Maroto F, Pozzi C., Salamini F, et al The barley *Hooded* mutation caused by a duplication in a homeobox gene intron. Nature 1995; 374: 727–730. 10.1038/374727a0 7715728

[18] Watkins AE, Ellerton S. Variation and genetics of the awn in *Triticum*. J Genet. 1940; 40: 243–270.

[19] McIntosh RA, Hart GE, Devos KM, Gale MD, Rogers WJ. Catalogue of gene symbols for wheat. Proc 9th Int Wheat Genet Symp. Saskatoon 1998; 5: 235.

[20] Sears E.R. The Aneuploids of Common Wheat. Missouri Agr Exp Stn Res Bull. 1954; 572.

[21] Sourdille P, Cadalen T, Gay G, Gill B, Bernard M. Molecular and physical mapping of genes affecting awning in wheat. Plant Breed. 2002; 121: 320–324.

[22] Kato K, Miura H, Akiyama M, Kuroshima M, Sawada S. RFLP mapping of the three major genes, *Vrn1*, *Q* and *B1*, on the long arm of chromosome 5A of wheat. Euphytica 1998; 101: 91–95.

[23] Gervais L, Dedryver F, Morlais JY, Bodusseau V, Negre S, Bilous M, et al Mapping of quantitative trait loci for field resistance to Fusarium head blight in an European winter wheat. Theor Appl Genet. 2003; 106: 961–970. 10.1007/s00122-002-1160-5 12671743

[24] Bariana HS, Parry N, Barclay IR, Loughman R, McLean RJ, Shankar M, et al Identification and characterization of stripe rust resistance gene *Yr34* in common wheat. Theor Appl Genet. 2006; 112: 1143–1148. 10.1007/s00122-006-0216-3 16435125

[25] Mackay IJ, Bansept-Basler P, Barber T, Bentley AR, Cockram J, Gosman N, et al An eight-parent multiparent advanced generation intercross population for winter-sown wheat: creation, properties and first results. G3 (Bethesda) 2014; 4: 1603–1610. 25237112 10.1534/g3.114.012963 PMC4169152

[26] Iehisa JCM, Ohno R, Kimura T, Enoki H, Nishimura S, Okamoto Y, et al A high-density genetic map with array-based markers facilitates structural and quantitative trait locus analyses of the common wheat genome. DNA Res. 2014; 21: 555–567. 10.1093/dnares/dsu020 24972598 PMC4195500

[27] Kobayashi F, Takumi S, Handa H. Identification of quantitative trait loci for ABA responsiveness at the seedling stage associated with ABA-regulated gene expression in common wheat. Theor Appl Genet. 2010; 121: 629–641. 10.1007/s00122-010-1335-4 20401645

[28] Takenaka S, Nitta M, Kawahara T, Nasuda S. “Core-collection” Project of the National BioResource Project-Wheat, Japan: 2013 Progress report. Wheat Inf Serv. 2014; 118: 23–24. Available from: http://shigen.nig.ac.jp/ewis/article/issuePage.do?issueNo=118.

[29] International Wheat Genome Sequencing Consortium. A chromosome-based draft sequence of the hexaploid bread wheat (*Triticum aestivum*) genome. Science 2014; 345: 1251788 10.1126/science.1251788 25035500

[30] Kofler R, Schlötterer C, Lelley T. SciRoKo: A new tool for whole genome microsatellite search and investigation. Bioinformatics 2007; 23: 1683–1685. 10.1093/bioinformatics/btm157 17463017

[31] Untergasser A, Nijveen H, Rao X, Bisseling T, Geurts R, Leunissen JAM. Primer3Plus, an enhanced web interface to Primer3. Nucleic Acids Res. 2007; 35: W71–W74. 10.1093/nar/gkm306 17485472 PMC1933133

[32] Hori K, Kobayashi T, Shimizu A, Sato K, Kawasaki S. Efficient construction of high-density linkage map and its application to QTL analysis in barley. Theor Appl Genet. 2003; 107: 806–813. 10.1007/s00122-003-1342-9 12838391

[33] Lorieux M. Fast and efficient computation of genetic linkage maps. Mol Breed. 2012; 30: 1231–1235.

[34] Voorrips RE. MapChart: Software for the graphical presentation of linkage maps and QTLs. J Hered. 2002; 93: 77–78. 12011185 10.1093/jhered/93.1.77

[35] Kobayashi F, Wu J, Kanamori H, Tanaka T, Katagiri S, Karasawa W, et al A high-resolution physical map integrating an anchored chromosome with the BAC physical maps of wheat chromosome 6B. BMC Genomics 2015; 16:595 10.1186/s12864-015-1803-y 26265254 PMC4534020

[36] Mayer KFX, Martis M, Hedley PE, Šimková H, Liu H, Morris JA, et al Unlocking the barley genome by chromosomal and comparative genomics. Plant Cell 2011; 23: 1249–1263. 10.1105/tpc.110.082537 21467582 PMC3101540

[37] Sourdille P, Singh S, Cadalen T, Brown-Guedira GL, Gay G, Qi L, et al Microsatellite-based deletion bin system for the establishment of genetic-physical map relationships in wheat (*Triticum aestivum* L.). Funct Integr Genomics. 2004; 4: 12–25. 10.1007/s10142-004-0106-1 15004738

[38] Kosuge K, Watanabe N, Kuboyama T, Melnik VM, Yanchenko VI, Rosova MA, et al Cytological and microsatellite mapping of mutant genes for spherical grain and compact spikes in durum wheat. Euphytica 2008; 159: 289–296.

[39] Cao L, Hayashi K, Tokui M, Mori M, Miura H, Onishi K. Detection of QTLs for traits associated with pre-harvest sprouting resistance in bread wheat (*Triticum aestivum* L.). Breed Sci. 2016; 66: 260–270. 10.1270/jsbbs.66.260 27162497 PMC4785003

[40] Luo MC, Gu YQ, You FM, Deal KR, Ma Y, Hu Y, et al A 4-gigabase physical map unlocks the structure and evolution of the complex genome of *Aegilops tauschii*, the wheat D-genome progenitor. Proc Natl Acad Sci USA. 2013; 110: 7940–7945. 10.1073/pnas.1219082110 23610408 PMC3651469

[41] Takahashi R, Yamamoto J, Yasuda S, Itano Y. Inheritance and linkage studies in barley. Ber Ohara Inst Landwirtschaftliche Forsch. 1953; 10: 29–53.

[42] Roig C, Pozzi C, Santi L, Müller J, Wang Y, Stile MR, et al Genetics of barley *Hooded* suppression. Genetics 2004; 167: 439–448. 15166167 10.1534/genetics.167.1.439 PMC1470836

[43] Takumi S, Kosugi T, Murai K, Mori N, Nakamura C. Molecular cloning of three homoeologous cDNAs encoding orthologs of the maize KNOTTED1 homeobox protein from young spikes of hexaploid wheat. Gene 2000; 249: 171–181. 10831851 10.1016/s0378-1119(00)00164-5

[44] Morimoto R, Kosugi T, Nakamura C, Takumi S. Intragenic diversity an functional conservation of the three homoeologous loci of the *KN1*-type homeobox gene *Wknox1* in common wheat. Plant Mol Biol. 2005; 57: 907–924. 10.1007/s11103-005-3247-2 15952073

