# Supplementary material for: Three dominant awnless genes in common wheat: Fine mapping, interaction and contribution to diversity in awn shape and length
Source: PLoS One. 2017 Apr 24;12(4):e0176148. doi: 10.1371/journal.pone.0176148 (PMC5402986; doi:10.1371/journal.pone.0176148)
Supplement: S1 Fig — First, BlastN searches of reads/contig sequences of the markers were performed against the wheat survey genome sequence. The aligned sequences were searched for PstI restriction sites for development of CAPS markers. If no PstI sites were found, SSR motifs were searched and primers were designed to amplify these motifs. If no SSR motifs were present in the genomic sequence and if the read/contig sequence was derived from M808, SNPs and indels were searched because the genome sequence was derived from CS. In total, 12 markers were developed and used for further analyses. (PDF) [file pone.0176148.s001.pdf]

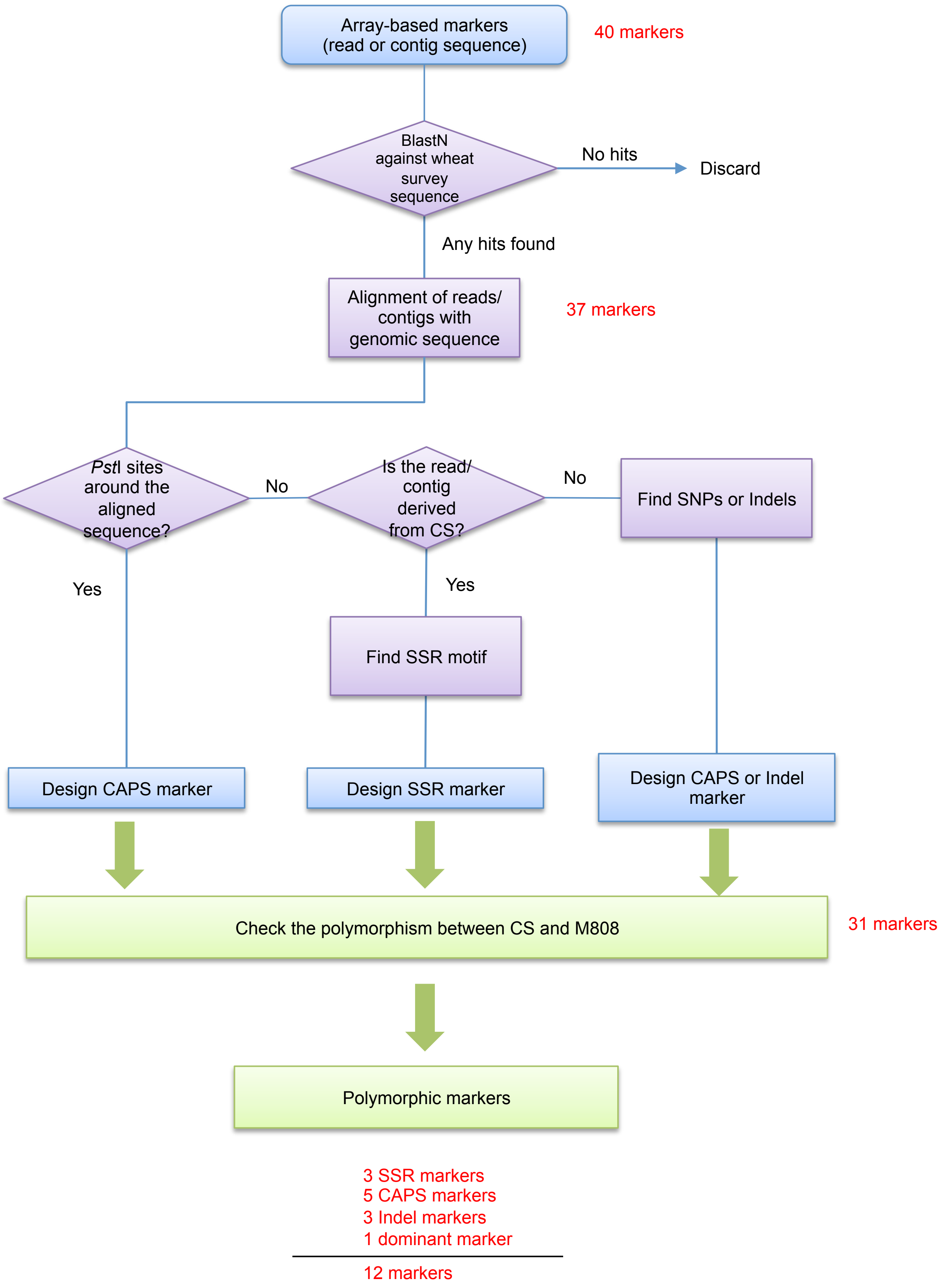

S1 Fig. Schematic of conversion of array-based markers to PCR-based markers. First, BlastN searches of reads/contig sequences of the markers were performed against the wheat survey genome sequence. The aligned sequences were searched for *Pst*I restriction sites for development of CAPS markers. If no *Pst*I sites were found, SSR motifs were searched and primers were designed to amplify these motifs. If no SSR motifs were present in the genomic sequence and if the read/contig sequence was derived from M808, SNPs and indels were searched because the genome sequence was derived from CS. In total, 12 markers were developed and used for further analyses.
