# Supplementary material for: Three dominant awnless genes in common wheat: Fine mapping, interaction and contribution to diversity in awn shape and length
Source: PLoS One. 2017 Apr 24;12(4):e0176148. doi: 10.1371/journal.pone.0176148 (PMC5402986; doi:10.1371/journal.pone.0176148)
Supplement: S2 Fig — Orthologs of rice DL (accession: Q76EJ0, OsDL) in Brachypodium distachyon (XP_010228969.1, BdDL), barley (BAK04901.1, HvDL), and the A-genome (306676_AA1011910.1, TaDL-A), B-genome (321049_AA1054410.1, TaDL-B) and D-genome of wheat (343031_AA1127860.1, TaDL-D) were aligned. Conserved amino acid residues are indicated by asterisks and the YABBY domain by the orange bar. (PDF) [file pone.0176148.s002.pdf]

```
OsDL      -----MDLVSPSEHLCYVRCTYCNTVLA--VGVPCKRLMDTVTVKCGHCNNLSFLSPRPP-MVQPLSPTD--
BdDL      MQQSSMDLVSPSEHLCYVRCTYCNTVLA--VGVPCKRLMDTVTVKCGHCNNLSFLSPRPPPMVQPLSPNDHH
TaeDL-B   MQ--SMDLVSPSEHLCYVRCTYCNTVLA--VGVPCKRLMDTVTVKCGHCNNLSFLSPRPPPMVQPLSPNDHH
TaeDL-A   MQ--SMDLVSPSEHLCYVRCTYCNTVLA--VGVPCKRLMDTVTVKCGHCNNLSFLSPRPPPMVQPLSPNDHH
TaeDL-D   MQ--SMDLVSPSEHLCYVRCTYCNTVLALQVGVPCKRLMDTVTVKCGHCNNLSFLSPRPPPMVQPLSPNDHH
HvDL      MQ--SMDLVSPSEHLCYVRCTYCNTVLALQVGVPCKRLMDTVTVKCGHCNNLSFLSPRPPPMVQPLSPNDHH
               ***********************  ****************************** ******* *

OsDL      HPLGPFQGPCTDCRRNQPLP-LVSPTSNEGSPRAPFVVKPPEKKHRLPSAYNRFMREEIQRIKAAKPDIPHR
BdDL      HPMGPFQG-CTDCRRNQPLPPLASPTSSDASPRAPFVVKPPEKKHRLPSAYNRFMREEIQRIKAAKPDIPHR
TaeDL-B   HPMGPFQG-CTDCRRNQPLPPLASPTSSDASPRAPFVVKPPEKKHRLPSAYNRFMREEIQRIKAAKPDIPHR
TaeDL-A   HPMGPFQG-CTDCRRNQPLPPLASPTSSDASPRAPFVVKPPEKKHRLPSAYNRFMREEIQRIKAAKPDIPHR
TaeDL-D   HPMGPFQG-CTDCRRNQPLPPLASPTSSDASPRAPFVVKPPEKKHRLPSAYNRFMREEIQRIKAAKPDIPHR
HvDL      HPMGPFQG-CTDCRRNQPLPPLASPTSSDASPRAPFVVKPPEKKHRLPSAYNRFMREEIQRIKAAKPDTPHR
          ** ***** *********** * ****   ************************************** ***

OsDL      EAFSMAAKNWAKCDPRCSSTVSTSNSNPEPR-VVAAPIPHQERANEQVVESFDIFKQMERSG
BdDL      EAFSMAAKNWAKCDPRCSSAVSASNSTSEPRSVVVPSPQLQERSNEQVVESFDIFKQMERSG
TaeDL-B   EAFSMAAKNWAKCDPRCSSTVSASNSAPEPR-IVVPGPQLQERATEQVVESFDIFKQMERST
TaeDL-A   EAFSMAAKNWAKCDPRCSSTVSASNSAPEPR-IVVPGPQLQERATEQVVESFDIFKQMERSA
TaeDL-D   EAFSMAAKNWAKCDPRCSSTVSASNSAPEPR-IIVPGPQLQERATEQVVESFDIFKQMERSA
HvDL      EAFSMAAKNWAKCDPRCSSTVSTSNSASEPR-IVVPGP--QERATEQVVESFDIFKQMERSA
          ******************* ** ***  ***         ***  ****************
```

S2 Fig. Alignment of DL and related protein sequences. Orthologs of rice DL (accession: Q76EJ0, OsDL) in *Brachypodium distachyon* (XP_010228969.1, BdDL), barley (BAK04901.1, HvDL), and the A-genome (306676_AA1011910.1, TaDL-A), B-genome (321049_AA1054410.1, TaDL-B) and D-genome of wheat (343031_AA1127860.1, TaDL-D) were aligned. Conserved amino acid residues are indicated by asterisks and the YABBY domain by the orange bar.
