# Supplementary material for: Three dominant awnless genes in common wheat: Fine mapping, interaction and contribution to diversity in awn shape and length
Source: PLoS One. 2017 Apr 24;12(4):e0176148. doi: 10.1371/journal.pone.0176148 (PMC5402986; doi:10.1371/journal.pone.0176148)
Supplement: S3 Fig — Awn length at the top (A) and middle (B) of the spike in the hexaploid wheat core collection. Subspecies of T. aestivum are indicated by different colors. Data are means ± SD. (PDF) [file pone.0176148.s003.pdf]

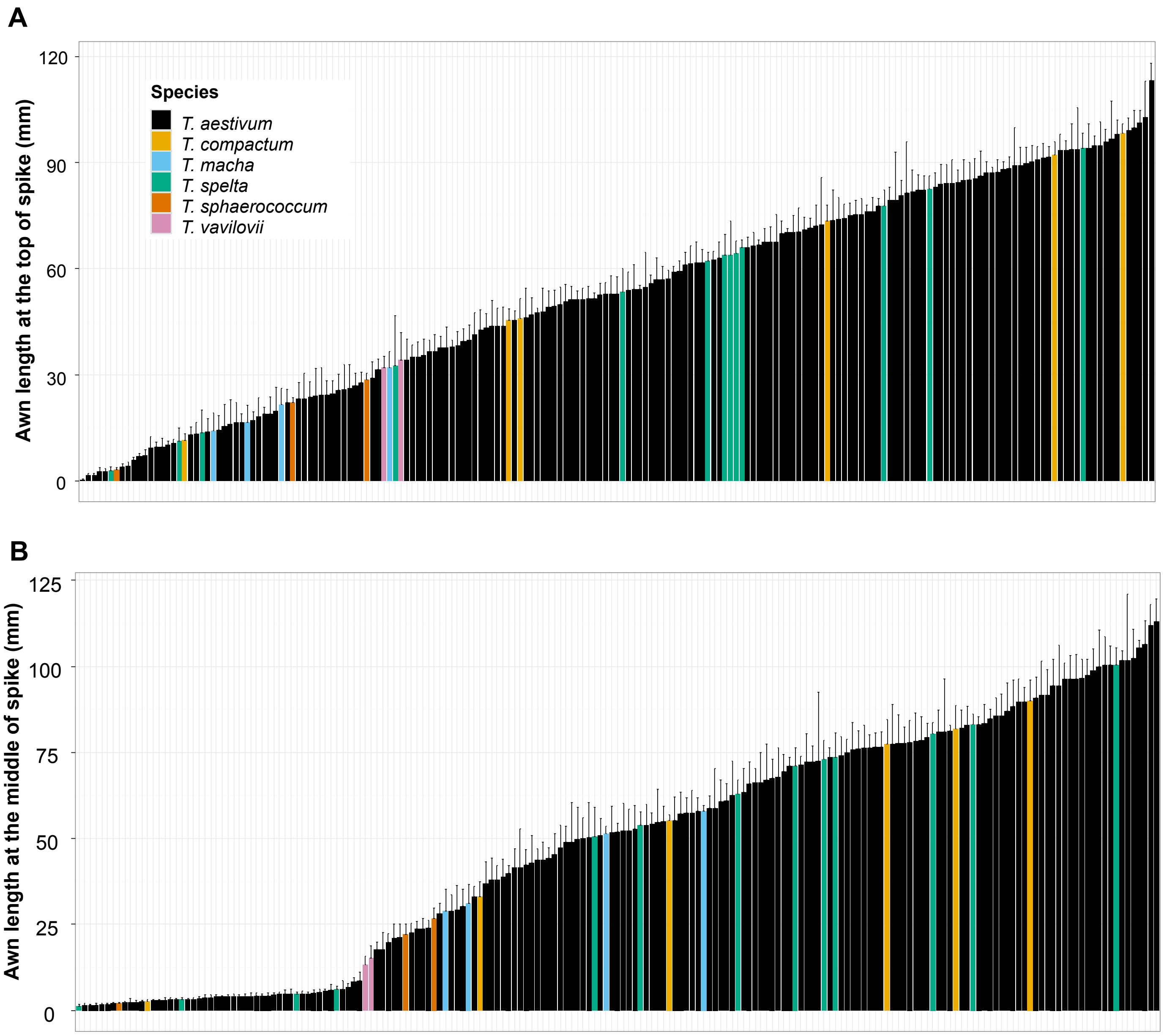

S3 Fig. Variation in awn length of the 189 hexaploid wheat varieties. Awn length at the top (A) and middle (B) of the spike in the hexaploid wheat core collection. Subspecies of *T. aestivum* are indicated by different colors. Data are means ± SD.
