# Supplementary material for: Three dominant awnless genes in common wheat: Fine mapping, interaction and contribution to diversity in awn shape and length
Source: PLoS One. 2017 Apr 24;12(4):e0176148. doi: 10.1371/journal.pone.0176148 (PMC5402986; doi:10.1371/journal.pone.0176148)
Supplement: S4 Fig — Box and whisker plots for awn length at the top (A) and middle (B) of the spike in the hexaploid wheat lines are grouped by geographical origin. (PDF) [file pone.0176148.s004.pdf]

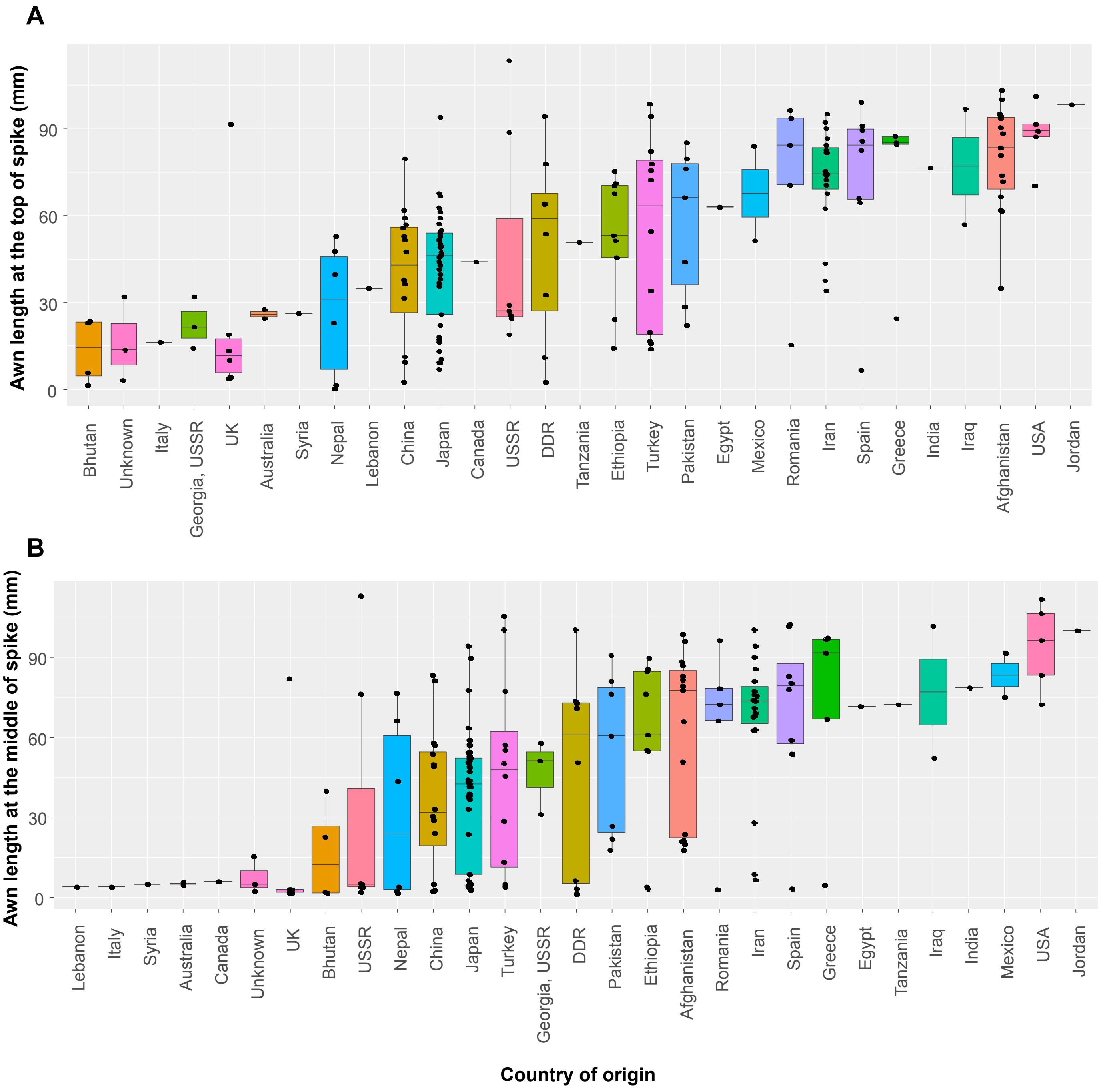

S4 Fig. Awn length of the hexaploids according to geographical origin. Box and whisker plots for awn length at the top (A) and middle (B) of the spike in the hexaploid wheat lines are grouped by geographical origin.
