# Supplementary material for: Three dominant awnless genes in common wheat: Fine mapping, interaction and contribution to diversity in awn shape and length
Source: PLoS One. 2017 Apr 24;12(4):e0176148. doi: 10.1371/journal.pone.0176148 (PMC5402986; doi:10.1371/journal.pone.0176148)
Supplement: S5 Fig — The linkage map was constructed using 161 RILs with known genotypes at the Hd, B1 and B2 loci to confirm the locations of the PCR-based markers developed. SSR markers were also included. (PDF) [file pone.0176148.s005.pdf]

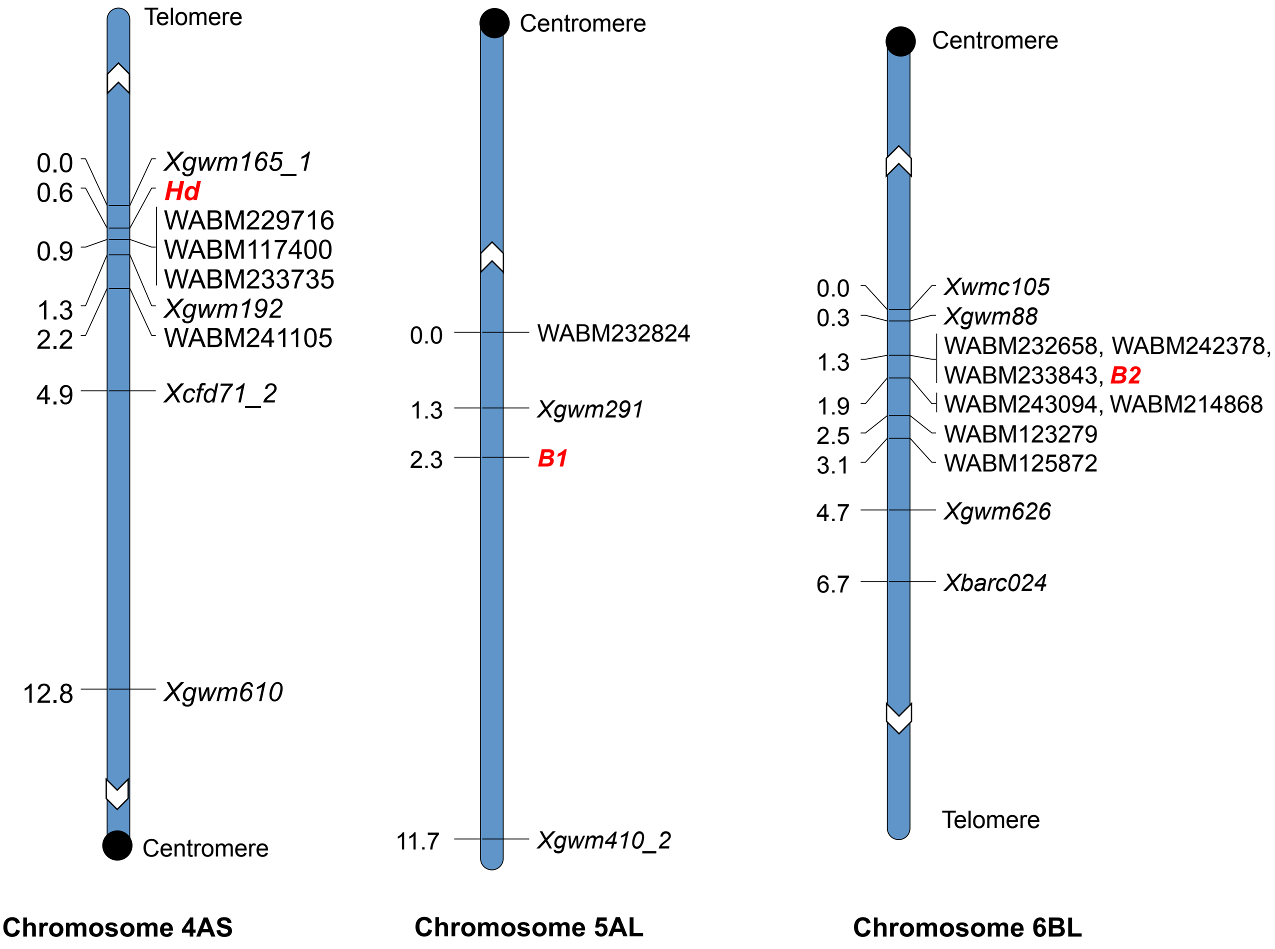

S5 Fig. Linkage map constructed using PCR-based markers. The linkage map was constructed using 161 RILs with known genotypes at the *Hd*, *B1* and *B2* loci to confirm the locations of the PCR-based markers developed. SSR markers were also included.
