# Supplementary material for: Three dominant awnless genes in common wheat: Fine mapping, interaction and contribution to diversity in awn shape and length
Source: PLoS One. 2017 Apr 24;12(4):e0176148. doi: 10.1371/journal.pone.0176148 (PMC5402986; doi:10.1371/journal.pone.0176148)
Supplement: S6 Fig — The physical map of chromosome 5A (left) and genetic maps (middle and right) were compared to locate the B1 locus on the physical map. Arrows indicate the break point of the chromosome deletion lines obtained by Endo and Gill. SSR markers mapped to the physical map of chromosome 5A were used to locate the B1 locus. (PDF) [file pone.0176148.s006.pdf]

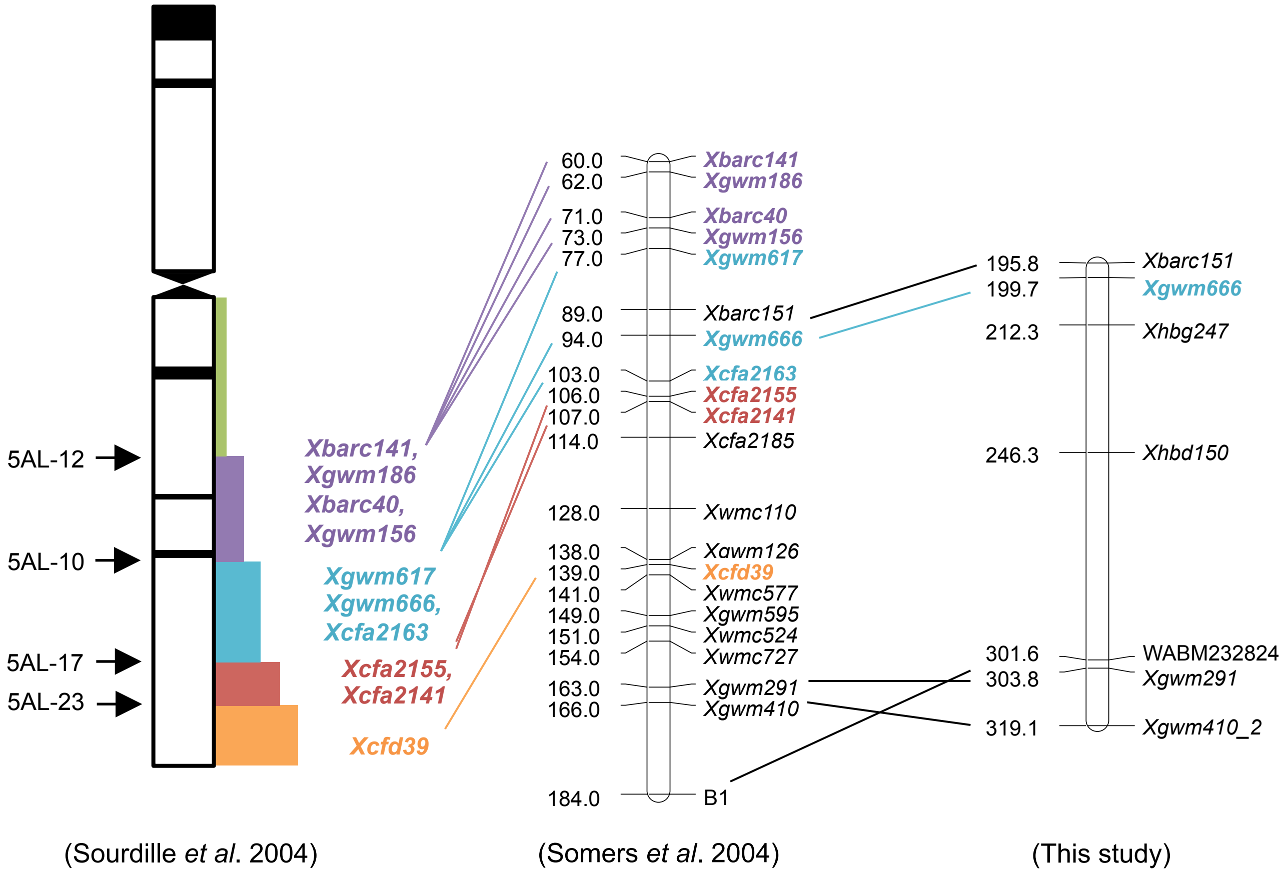

S6 Fig. Comparative map indicating the location of the *B1* locus. The physical map of chromosome 5A (left) and genetic maps (middle and right) were compared to locate the *B1* locus on the physical map. Arrows indicate the break point of the chromosome deletion lines obtained by Endo and Gill [45]. SSR markers mapped to the physical map of chromosome 5A were used to locate the *B1* locus.

45. Endo TR, Gill BS. The deletion stocks of common wheat. J Hered. 1996; 87: 295–307.
