# Supplementary material for: Three dominant awnless genes in common wheat: Fine mapping, interaction and contribution to diversity in awn shape and length
Source: PLoS One. 2017 Apr 24;12(4):e0176148. doi: 10.1371/journal.pone.0176148 (PMC5402986; doi:10.1371/journal.pone.0176148)
Supplement: S2 Table — *These two SSR markers have been previously described (http://wheat.pw.usda.gov/GG3/). (PDF) [file pone.0176148.s008.pdf]

S2 Table. Primers used in this study

Primer name	Sequence (5’ -> 3’)	Restriction enzyme	Chromosome
WABM233735PCR	ATCTCGTGACCCCAGTACCT<br>GCTGGAACCGGCAAAATCAG	*Pst*I	4A
WABM241105PCR	TGGCCAAGCTTTCACTTCCA<br>GTGGAAGTAACGCGGGAAGA	*Pst*I	4A
WABM229716PCR	GACGATTTGCTTTCCCCGTC<br>ACCATAAAAAGAAGGGAAAAGGCT	-<br>(SSR)	4A
WABM117400PCR	AAAGGTCGGAGGTGACAAGC<br>GCGCACTAACCGGGGAATAA	-<br>(SSR)	4A
*Xgwm192**	GGTTTTCTTTCAGATTGCGC<br>CGTTGTCTAATCTTGCCTTGC	-<br>(SSR)	4A
WABM232824PCR	ACAAGCTCAAGCTGGTCCAT<br>TGGCACAGGTGCTATTGGTA	-<br>(Indel)	5A
*Xgwm291**	CATCCCTACGCCACTCTGC<br>AATGGTATCTATTCCGACCCG	-<br>(SSR)	5A
WABM232658PCR	AAGTTCGCCTCTTCACCAGT<br>TCTGCCCCTACATCTGTTGC	-<br>(Indel)	6B
WABM242378PCR	GTAGATATATGGCATGCAAAAGCCA<br>TTCAGATTGAATATTAGTACGCTGAAA	*Pst*I	6B
WABM233843PCR	AAAGTTCACGACAGCGGAGT<br>AACGAGAAGATGACCCCAGC	*Pst*I	6B
WABM243094PCR	ACCACAGAAAAACGCATGCC<br>TGGGTAGTCTTTTGCTGCCT	*Bst*PI	6B
WABM214868PCR	GGGTGCCTGAACATTGATGC<br>CCCCAAGTGCTGTCGTGTAT	-<br>(SSR)	6B
WABM125872PCR	ACTTAGTCAACATGCCCCACA<br>ACCTCACTTGCGACATTGCT	-	6B
WABM123279PCR	CTCAAGCTGGTGGCAAGAGA<br>GACTTTGTTTGGACGCCACC	-<br>(Indel)	6B

*These two SSR markers have been previously described (http://wheat.pw.usda.gov/GG3/).
