# Supplementary material for: Three dominant awnless genes in common wheat: Fine mapping, interaction and contribution to diversity in awn shape and length
Source: PLoS One. 2017 Apr 24;12(4):e0176148. doi: 10.1371/journal.pone.0176148 (PMC5402986; doi:10.1371/journal.pone.0176148)
Supplement: S4 Table — A BlastP search was performed against the EnsemblPlants Triticum aestivum protein database. (PDF) [file pone.0176148.s010.pdf]

S4 Table. Orthologs of genes involved in awn development in wheat

Gene	Locus	Orthologs in wheat	Similarity (%)
Lks2	MLOC_74469 (7HL)	TRIAE_CS42_7AL_TGACv1_557584_AA1783580	98.6
		TRIAE_CS42_7BL_TGACv1_577937_AA1886410	97.4
		TRIAE_CS42_7DL_TGACv1_602657_AA1964260	98.6
LABA1	Os04g0518800	TRIAE_CS42_2AL_TGACv1_094044_AA0291560	78.9
		TRIAE_CS42_2BL_TGACv1_129499_AA0386210	80.1
		TRIAE_CS42_2DL_TGACv1_158365_AA0516710	78.5
DL	Os03g0215200	TRIAE_CS42_4AS_TGACv1_306676_AA1011910	89.0
		TRIAE_CS42_4BL_TGACv1_321049_AA1054410	88.4
		TRIAE_CS42_4DL_TGACv1_343031_AA1127860	88.5
OsETT2	Os01g0670800	TRIAE_CS42_3AL_TGACv1_195685_AA0652720	75.1
		TRIAE_CS42_3B_TGACv1_221306_AA0736770	75.0
		TRIAE_CS42_3DL_TGACv1_251881_AA0885700	75.6
SHL2	Os01g0527600	TRIAE_CS42_3B_TGACv1_223814_AA0787720	76.6
		TRIAE_CS42_3DL_TGACv1_249614_AA0852540	77.2
RAE2	Os08g0485500	TRIAE_CS42_6AL_TGACv1_471646_AA1512300	65.0
		TRIAE_CS42_6BL_TGACv1_499434_AA1582500	51.4
		TRIAE_CS42_6DL_TGACv1_527458_AA1704280	51.4
An-1	Os04g0350700	TRIAE_CS42_2AL_TGACv1_093921_AA0289390	54.5
		TRIAE_CS42_2BL_TGACv1_129357_AA0380060	52.2
		TRIAE_CS42_2DL_TGACv1_160046_AA0545980	53.3
HvKnox3	CAA58503.1	TRIAE_CS42_4AL_TGACv1_291876_AA0996890	97.0
		TRIAE_CS42_4BS_TGACv1_328386_AA1087310	97.5
		TRIAE_CS42_4DS_TGACv1_361480_AA1168780	97.3

A BlastP search was performed against the EnsemblPlants *Triticum aestivum* protein database.
